# Molecular profiling and sequential somatic mutation shift in hypermutator tumours harbouring POLE mutations

**DOI:** 10.1038/s41598-018-26967-4

**Published:** 2018-06-07

**Authors:** Keiichi Hatakeyama, Keiichi Ohshima, Takeshi Nagashima, Shumpei Ohnami, Sumiko Ohnami, Masakuni Serizawa, Yuji Shimoda, Koji Maruyama, Yasuto Akiyama, Kenichi Urakami, Masatoshi Kusuhara, Tohru Mochizuki, Ken Yamaguchi

**Affiliations:** 1 Medical Genetics Division, Shizuoka Cancer Center Research Institute, Sunto-gun, Shizuoka, 411-8777 Japan; 2 Cancer Diagnostics Research Division, Shizuoka Cancer Center Research Institute, Sunto-gun, Shizuoka, 411-8777 Japan; 3 SRL Inc., Shinjuku-ku, Tokyo, 163-0409 Japan; 4 Drug Discovery and Development Division, Shizuoka Cancer Center Research Institute, Sunto-gun, Shizuoka, 411-8777 Japan; 5 Experimental Animal Facility, Shizuoka Cancer Center Research Institute, Sunto-gun, Shizuoka, 411-8777 Japan; 6 Immunotheraphy Division, Shizuoka Cancer Center Research Institute, Sunto-gun, Shizuoka, 411-8777 Japan; 7 Regional Resource Division, Shizuoka Cancer Center Research Institute, Sunto-gun, Shizuoka, 411-8777 Japan; 8 Shizuoka Cancer Center, Sunto-gun, Shizuoka, 411-8777 Japan

## Abstract

Defective DNA polymerase ε (POLE) proofreading leads to extensive somatic mutations that exhibit biased mutational properties; however, the characteristics of *POLE*-mutated tumours remain unclear. In the present study, we describe a molecular profile using whole exome sequencing based on the transition of somatic mutations in 10 *POLE*-mutated solid tumours that were obtained from 2,042 Japanese patients. The bias of accumulated variations in these mutants was quantified to follow a pattern of somatic mutations, thereby classifying the sequential mutation shift into three periods. During the period prior to occurrence of the aberrant *POLE*, bare accumulation of mutations in cancer-related genes was observed, whereas *PTEN* was highly mutated in conjunction with or subsequent to the event, suggesting that *POLE* and *PTEN* mutations were responsible for the development of *POLE*-mutated tumours. Furthermore, homologous recombination was restored following the occurrence of PTEN mutations. Our strategy for estimation of the footprint of somatic mutations may provide new insight towards the understanding of mutation-driven tumourigenesis.

## Introduction

Large-scale genomic studies have revealed the rare occurrence of a diversity of mutation frequency and somatic hypermutation in specific tumours, which are termed ‘hypermutators’^1–3^. This mutator effect in colorectal and endometrial cancers is occasionally accompanied by mutations in the exonuclease domain of DNA polymerase epsilon (POLE)^2–4^. The *POLE* gene is responsible for the replication of a leading DNA strand^5–7^; hence, the defective proofreading consequent to its mutation is considered to generate extensive somatic mutations that constitute a distinct mutation spectrum^8^. In particular, p.P286R and p.V411L substitutions lead to loss of POLE proofreading activity^8,9^. Furthermore, mutations in the exonuclease domain of POLE are observed in microsatellite stable (MSS) tumours that are characterised mainly by a biased prevalence of TCT > TAT and TCG > TTG mutation patterns^10^. Although the characteristics of *POLE* mutation are well understood, that of its associated tumours remains unclear, in large part because in previous reports, tumours harbouring this mutant have been specifically eliminated from the interpretation of genomic analysis as an anomalous case^2,3^.

Nearly three decades ago, Vogelstein *et al*. proposed that sequential mutations in specific oncogenes and tumour suppressor genes (TSGs) lead to tumourigenesis^11^ based on the two-hit hypothesis formulated by Knudson^12^. As such mutations are observed in many tumours, this concept is still widely accepted. However, it is difficult to directly trace the sequential shift of somatic mutations in a solid tumour that occurs in a patient. Accordingly, in the majority of large-scale genomic studies, the mutation footprint is predicted only from an endpoint of base substitutions in genes determined after lesion resection.

The molecular profile in a normal cell is dramatically changed upon the occurrence of mutation in a specific gene as an early event of tumourigenesis. In cultured human intestinal stem cells, artificial mutation of *APC*, *TP53* (also known as P53), and/or *KRAS* by CRISPR/Cas9 technology is sufficient to serve as a hallmark of tumour progression^13^. Hence, estimation of the period when key gene mutations arise and investigation of cancer-related genes that mutated prior to or concomitant with this event are of considerable importance to the understanding of mutation-driven tumourigenesis.

In the current study, to reveal the molecular profile and sequential mutation shift of hypermutators harbouring a POLE mutant, we investigated the mutation pattern and gene expression based on biased base substitutions. *POLE*-mutated samples were first extracted from more than 2,000 Japanese patients with cancer followed by confirmation of whether these tumours possessed characteristics similar to those reported in previous studies. We next revealed that the susceptibility to effects of the POLE mutant differed according to gene based on the pattern of base substitutions. The sequential mutation shift of genes that accumulated in tumours with POLE mutants were then classified into three periods. *POLE* and phosphatase and tensin homolog (*PTEN*) mutations were therefore predicted to influence early tumourigenesis. Furthermore, defective homologous recombination (HR) was restored in comparison with that in common hypermutator tumours, and somatic mutations were found to accumulate in pathways associated with hypoxia-inducible factor, implying their role in maintaining HR repair activity. Overall, our analysis using the biased base substitution paradigm allows an accelerated understanding of mutation-driven tumourigenesis.

## Results

### Molecular subtypes of hypermutators

**Figure 1:**
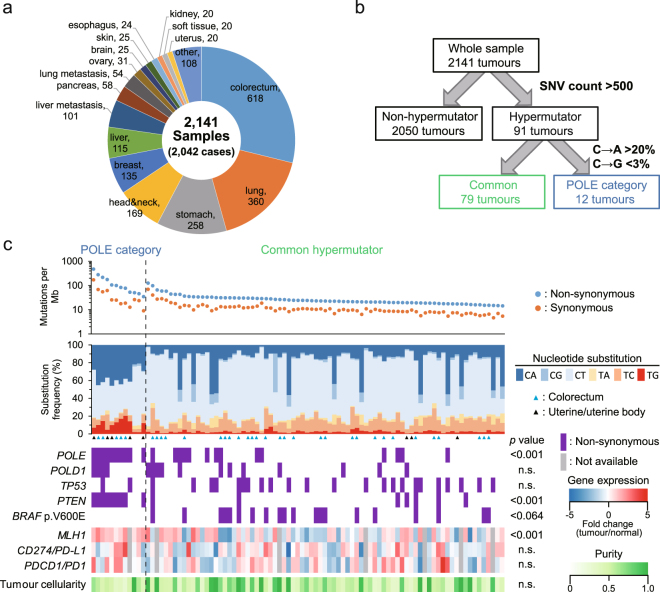
Sample classification and mutation spectra. (**a**) Distribution of tumour types included in this analysis. The ‘other’ group contains multiple tumour types that comprise less than 20 samples. (**b**) All samples were stratified into three groups according to the nucleotide substitution frequency and pattern. SNV, single nucleotide variation. (**c**) Mutation frequencies (vertical axis, top panel) plotted for POLE-category and common hypermutator (horizontal axis) tumours. Nucleotide substitutions are shown in the middle panel. Tumours of the colorectum and uterine/uterine body are indicated by red and black arrowheads, respectively. Mutation pattern, expression profile, and tumour cellularity are represented in the bottom panel. Gene expression is analysed and coloured based on fold change (tumour/normal). In cases where tumour type created difficulty with respect to dissection of the normal sample, a grey-filled rectangle (not available) is shown in the profile. The *p* values of mutation and expression/cellularity are calculated using Fisher’s exact test and Welch’s *t*-test, respectively. n.s., not significant.

We performed whole exome sequencing (WES) using 2,141 solid tumour samples derived from 2,042 patients with cancer. All samples collected from our single hospital were comprised of multiple tissues, among which colorectal, lung, and stomach cancers occupied 58% of the whole content (Fig. 1a). The tumours exceeding 500 counts of non-synonymous single nucleotide variation (SNV) were grouped as hypermutators (Fig. 1b). A total of 91 samples (4.3%) belonged to this group, yielding a frequency that was similar to that in a previous large-scale analysis^1^. The hypermutators were then classified into two subtypes according to nucleotide substitution frequency and pattern^2^. The tumours that met the criteria were defined as POLE category in this study, whereas the remaining hypermutators were categorised as common.

### Landscape of POLE category and hypermutators

To further clarify the differences between the two subtypes, gene expression profiling (GEP) and an estimation of tumour cellularity were performed along with mutation analysis (Fig. 1c). The number of mutations (per Mb) in POLE-category tumours was significantly higher than in common hypermutators (*p* = 1.14 × 10^−2^ by Welch’s *t*-test). Additionally, patients classified with POLE category tumours were significantly younger than those of with hypermutator tumours (Supplementary Fig. S1). In POLE category, tumours derived from the colorectum and uterus were dominant except in two samples of soft tissue that were dissected from the same patient with a diagnosis of melanoma. These samples without *POLE* mutations were checked for 30 mutational signatures^1^ using deconstructSigs^14^, indicating the presence of predominantly ultraviolet-induced Signature.7 (data not shown). To characterise POLE mutant tumours, the samples without *POLE* mutation were removed from consideration in this study. Consistent with a previous report^2^, accumulation of *PTEN* mutation and up-regulation of *MLH1* were observed in POLE-category tumours (Fig. 1c). No mutation of *BRAF* p.V600E was found; additionally, no differences of gene expression on immune checkpoint molecules (PD-L1 and PD1) or tumour cellularity were observed.

### Propensity of mutation in POLE-category tumours

**Figure 2:**
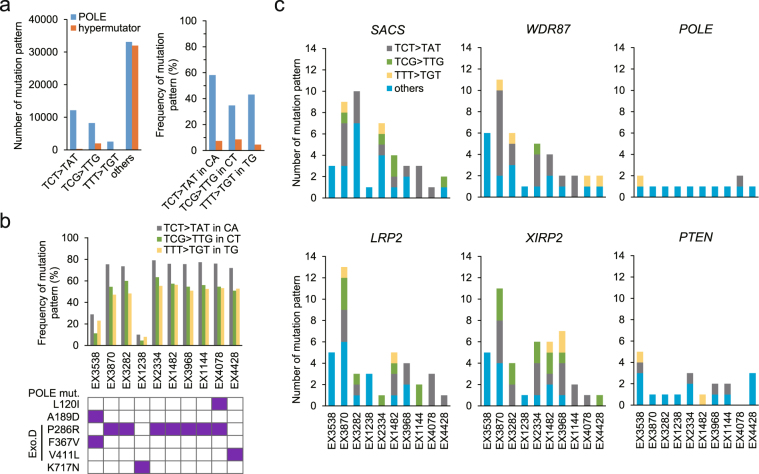
Propensity of mutation patterns in POLE-category tumours. (**a**) Number and frequency of mutation patterns in POLE-category and common hypermutator tumours. (**b**) Individual frequency of mutation patterns and amino acid substitution on POLE. The mutation is shown as purple-filled cells. Exo D, exonuclease domain. (**c**) Distribution of mutation patterns in *SACS*, *WDR87*, *LRP2*, and *XIRP2* genes that were specifically enriched for propensity mutations in the POLE category. *POLE* and *PTEN* are shown as known mutation-enriched genes in this category.

Although a common hypermutator is frequently characterised as having microsatellite instability (MSI), a tumour harbouring *POLE* mutations was described as belonging to MSS cancer type^4^. MSI testing was therefore performed in POLE-category tumours, indicating that all samples were MSS (Table 1). The exonuclease domain of *POLE* and/or *POLD1* was mutated in all samples of this category except those derived from soft tissue. The *POLE* p.V411L or p.P286R substitutions are known to generate a relatively large excess of C > A/T transversion in the specific three-letter base motifs (TCT and TCG) owing to defects in DNA proofreading^8,10^. A similar propensity was observed in the POLE-category group (Fig. 2a) and these were especially accumulated in tumours harbouring *POLE* p.P286R and p.V411L (Fig. 2b). Furthermore, T > G substitution tended to preferentially occur with neighbour thymines (i.e., TTT). These results indicated that *POLE* p.P286R and p.V411L generated a biased substitution depending on the surrounding sequence.

**Table 1:** POLE mutations in POLE-category tumours.

Substitution		No. of SNVs	MSI	Tissue
POLE	POLD1			
p.A189D, p.**F367V**	p.**R244H**	16632	MSS	uterus
p.K717N	p.**E318G**	6155	MSS	uterus
p.**P286R**	—	3686	MSS	uterine body
p.L120I, p.**P286R**	—	1846	MSS	uterus
p.**V411L**	—	1212	MSS	uterus
p.**P286R**	p.A706T	9782	MSS	colon (transverse)
p.**P286R**	p.**D402N**	7593	MSS	colon (ascending)
p.**P286R**	—	3491	MSS	colon (rectum)
p.**P286R**	—	2903	MSS	colon (rectum)
p.**P286R**	—	2699	MSS	colon (rectum)

The substitutions located in the exonuclease domain in POLE/POLD1 are represented in bold text. Microsatellite instability (MSI) was checked using PCR of five microsatellite markers (*BAT25*, *BAT26*, *NR21*, *NR24*, and *MONO27*) by an outsourcing company. SNV, single nucleotide variation (non-synonymous); MSS, microsatellite stable.

### Identification of variation-susceptible genes

To identify genes susceptible to POLE mutant effects, we counted the mutation patterns in individual tumours. The biased mutation patterns TCT > TAT, TCG > TTG, and TTT > TGT were highly accumulated in *SACS*, *LRP2*, *WDR87*, and *XIRP2* genes, whereas no accumulation of these patterns was observed in *POLE* and *PTEN* (Fig. 2c). In these genes, the biased mutation patterns were detected in all POLE category tumours with *POLE* p.P286R or p.V411L. To remove any concern of inherent bias derived from gene sequence, all patterns of three-letter bases in the exons of each gene were tallied and the occurrence frequency of mutation patterns was then estimated. Although these genes contained low frequencies of TCG sequence, a cytosine in this motif was highly mutated compared to other sites except in *POLE* and *PTEN* (Supplementary Fig. S2a). Furthermore, over 80% of TCG and TCT motifs evidenced the biased transversion; i.e., propensity mutation (Supplementary Fig. S2b). These results indicated that propensity mutation via *POLE* p.P286R or p.V411L was less affected by the appearance frequency of three-letter base sequences. Conversely, in a public repository (COSMIC, http://cancer.sanger.ac.uk/cosmic), the mutation patterns tended to exhibit a similar propensity (Supplementary Fig. S3a and S3b). In this study, *SACS*, *LRP2*, *WDR87*, and *XIRP2* were therefore evaluated as highly susceptible genes to POLE mutant status.

### Gene classification by POLE-category specific mutations

**Figure 3:**
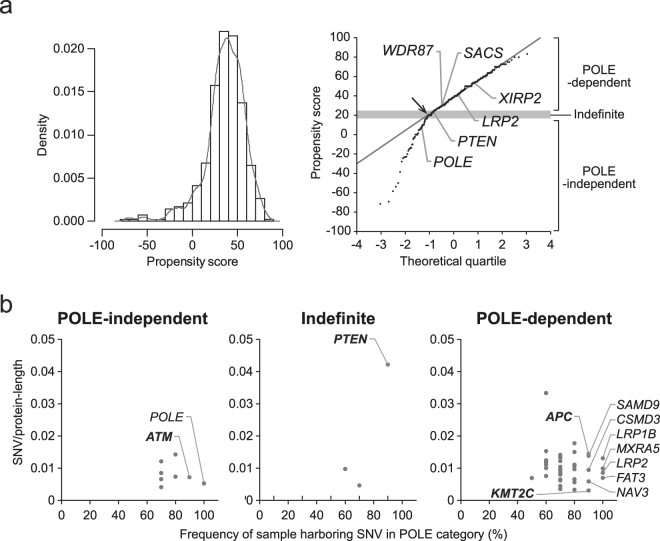
Classification of POLE-category-specific mutations into POLE-independent and -dependent mutations. (**a**) Frequency density (left) and quantile-quantile (Q-Q) plot (right) of propensity score (PS) in the gene harbouring POLE-category-specific mutations. Gene symbols correspond to Fig. 2c. Inflection point (IP) in the Q-Q plot is indicated by an arrow and is defined as the threshold of PS to separate genes possessing POLE-independent and -dependent mutations. The genes in the grey rectangle (confidence interval of IP) are classified in the group of indefinite mutation as a border for buffering. (**b**) Comparison of the mutation rates for protein coding regions between cancer-related genes in the classified groups. Gene symbols are represented when more than 80% of mutations could be classified as POLE category. Tumour suppressor genes are shown with a bold symbol.

Based on the mutation pattern, we found four genes that were highly mutated by the POLE mutant. To further identify other variation-susceptible genes, propensity score (PS) was determined and calculated using the frequency of mutation patterns in individual genes within POLE-category and common hypermutator tumours (see Methods, equations (1) and (2)). The PS based on the pattern frequency was independent of length of coding region. In particular, low PS indicates that a gene was mutated independently of a POLE mutant, whereas high PS specifies that the propensity mutation was accumulated dependently of the aberrant *POLE*. The workflow of classification using PS is shown in Supplementary Fig. S4. Briefly, candidates harbouring POLE-category specific mutations were first extracted prior to the calculation of PS to filter out extraneous mutated genes (listed in Supplementary Table S1). Then, low-mutation-number genes (SNV < 10) in the POLE-category group were removed, resulting in 414 genes isolated. The contribution of propensity sequence (TCT and TCG) appearance frequency in the isolated genes to PS was negligible (Supplementary Fig. S6a). Variant allele frequency (VAF) of propensity mutation patterns (TCT > TAT and TCG > TTG) was additionally calculated in these genes (Supplementary Fig. S5). Next, inflection point (IP) was derived from the distribution and quantile-quantile (Q-Q) plot of PS (Fig. 3a) based on segmented regression (standard linear model; break-point, 1; see Methods). In order to construct a confidence interval (CI) of IP, we employed a bootstrap method. Finally, genes in the CI of IP were classified in the indefinite period; the remaining low PS (<lower-CI) genes were determined as POLE-independent, and the high PS (≥upper-CI) genes were considered POLE-dependent. We expected that the four genes (*SACS*, *LRP2*, *WDR87*, and *XIRP2*) that were detected in all POLE category tumours with *POLE* p.P286R or p.V411L would exhibit high PS if this score reflected an accumulation of biased mutation patterns. Consistent with this supposition, these genes were grouped in the POLE-dependent category. This result suggested that the classification into three periods using PS was satisfactory to isolate genes susceptible to POLE mutant activity.

A common hypermutator is frequently characterised as having MSI. To confirm the influence of this status on PS, the mutation pattern in other MSI high/low samples was checked as well as in common hypermutators derived from colon and uterus tumours. Tumours with MSI high were extracted based on 30 mutational signatures^1^ assessed using deconstructSigs (Signature.6 (MSI) > 0.5)^14^. Accumulation of the POLE-specific mutation patterns (TCT > TAT, TCG > TTG, and TTT > TGT) was detected at less than 10% in all groups (Supplementary Fig. S6b). Moreover, PS is composed only of FMPs (propensity mutations/total mutations in each group, see Methods). We performed correlation analysis of all FMPs including MSI-high/low groups, demonstrating that no significant correlation was observed between FMPs (Supplementary Fig. S6c). These results indicated that the tumour MSI status had no influence on PS.

### Discovery of variation-susceptible tumour suppressor genes

Using PS based on the propensity of POLE mutant effects, POLE-category specific genes were classified according to the three periods. To estimate the impact of mutations generated by the POLE mutant on tumourigenesis, we analysed the substitution frequency of cancer-related genes. This gene set was curated in-house using multiple public databases (Supplementary Table S1). Additionally, TSGs and oncogenes were defined based on Vogelstein’s list^15^. No mutations of TSGs and oncogenes were observed in the POLE-independent period, whereas *PTEN*, *ATM*, *APC*, and *KMT2C* were mutated in both the indefinite and POLE-dependent periods (Fig. 3b). Notably, the non-synonymous mutation rate per protein-length of *PTEN* was higher than that of other TSGs.

### Validation of propensity mutations using public data

To validate the variation susceptibility of POLE-category specific genes, classification using PS was performed in the International Cancer Genome Consortium (ICGC) data set. The PS distribution that was similar to our study was classified into three periods to visualise sequential mutation shift (Supplementary Fig. S7a). The mutation accumulation in five genes (*POLE*, *PTEN*, *CSMD3*, *APC*, and *NAV3*) was consistent with our data set. In POLE-category tumours derived from ICGC data, *POLE* and *PTEN* accumulated POLE-independent mutations (Supplementary Fig. S7b). This result suggested that *POLE* and *PTEN* were almost unsusceptible to defective POLE function.

### Estimation of HR activity

**Figure 4:**
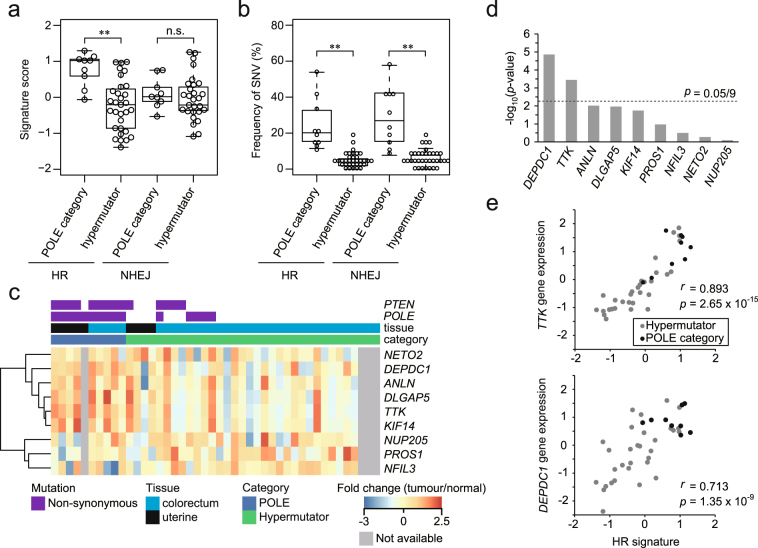
Expression profiles of homologous recombination (HR)-related genes in POLE-category tumours. Gene signature analysis (**a**) and mutation frequency (**b**) of HR and non-homologous end joining (NHEJ) genes between POLE-category and common hypermutator tumours. Frequency of SNV represents accumulation of mutations found in the related gene sets (52 and 27 genes in HR and NHEJ, respectively)^20,21^. All SNVs were counted in each sample. n.s., not significant; **p < 0.01. (**c**) Heat map of genes that were up-regulated in the reversal of HR deficiency. The column represents each sample in POLE category and common hypermutators derived from colon and uterus. In cases where tumour type created difficulty with respect to dissection of the normal sample, a grey-filled rectangle (not available) is shown in the profile. (**d**) Bar chart representing the significant genes that are compared between POLE-category and common hypermutator tumours in the heat map (**c**). To counteract the problem of multiple comparisons in the expression profile, *p*-value was adjusted with Bonferroni correction. (**e)** Correlation between HR signature and significantly up-regulated genes in the bar chart (**d**).

Aberrant PTEN often leads to dysfunction of HR^16–18^. The HR activity in POLE category tumours was first evaluated based on expression signatures. HR and non-homologous end joining (NHEJ) signatures were composed of five (*BRCA1*, *RAD51*, *BRCA2*, *RAD54L*, and *RAD52*) and four (*LIG4*, *PRKDC*, *XRCC4*, and *XRCC6*) genes that up-regulated activation of these functions, respectively^19^. The HR signature in POLE mutants was high in comparison with that in common hypermutator tumours (*p* = 6.06 × 10^−5^, Fig. 4a), whereas mutations of HR-/NHEJ-related genes^20,21^ were accumulated in POLE-category tumours (Fig. 4b), implying that HR activity in tumours harbouring defective POLE were elevated despite mutation accumulation in HR genes. To confirm the influence of MSI status, we compared these signatures and the frequency of SNV between MSI high/low samples in common hypermutators. MSI high were extracted using deconstructSigs^14^ (Signature.6 (MSI) > 0.5). No significant differences were observed between MSI high and MSI low subsets among common hypermutators (Supplementary Fig. S8a,b). Additionally, no significant impact of tissue distribution was found in POLE category and common hypermutator tumours (Supplementary Fig. S8c,d). These results indicated that MSI status and tissue distribution had no influence on HR-/NHEJ-signatures and mutations in common hypermutators. As PTEN-deficient tumours with mutations of HR-related genes are considered as a reversal of HR deficiency^22^, we therefore next investigated nine genes up-regulated in the process of HR repair restoration (in the previous report^22^, ≥0 score in ≥75% samples). Six genes (67%) tended to be highly expressed in POLE-category tumours, with *DEPDC1* and *TTK* genes in particular exhibiting significant differences in expression (Fig. 4c,d). Additionally, expression of *TTK*, which can increase HR repair^22^, was strongly positively correlated with HR signature (Fig. 4e). To further confirm whether *PTEN* mutated tumours restored HR repair deficiency, we investigated the relationship between *PTEN* mutation and HR-related genes (HR signature, *TTK* and *DEPDC1*) in colon and uterine tumours including non-hypermutators. These genes were found to be up-regulated in *PTEN* mutated tumours (Supplementary Fig. S9). These results suggested that POLE-category tumours with *PTEN* mutations restored HR repair deficiency through *TTK* overexpression.

### Pathway alteration in POLE-category tumours

**Figure 5:**
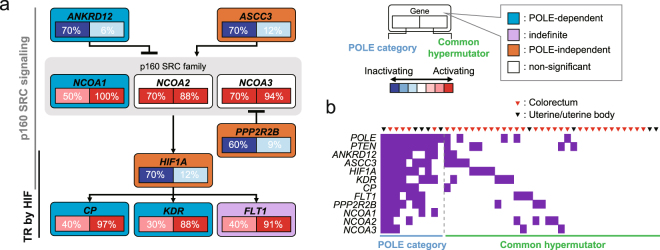
Pathway alterations in POLE-category tumours. (**a**) The p160 steroid receptor co-activator (SRC) signalling and transcriptional regulation (TR) by hypoxia inducible factor (HIF) pathways are altered through accumulation of somatic mutations. Alteration frequencies are expressed as a percentage of all cases. The genes that were significantly mutated between our study and public repository are represented. (**b**) Mutation patterns in the p160 SRC signalling and TR by HIF pathways.

To further characterise POLE-category tumours, we investigated pathways accumulating POLE-category-specific mutations between our data and ICGC data sets. Mutation accumulation in genes associated with transcriptional regulation (TR) by hypoxia inducible factor (HIF) and p160 steroid receptor co-activator (SRC) signalling pathways were observed in both data sets (Supplementary Fig. S10). In the two pathways, we identified eight genes that accumulated POLE-category-specific mutations (Fig. 5a and b). The pathways were linked via HIF1A, which is categorised as POLE-independent, and the frequency of mutation in the eight genes was similar to that in the ICGC data set (Supplementary Fig. S11). These results suggested that the two pathways linked via HIF1A were compromised by excess accumulation of POLE category mutations.

## Discussion

In tumours exhibiting a hypermutator phenotype, the presence of a subtype that possesses different characteristics has been revealed by a team of The Cancer Genome Atlas Network^2,3^. This subtype harbours POLE mutations and is predominantly found in colorectal and uterine tumours. We also identified hypermutators harbouring such mutations from the same tissue types that had been collected from more than 2,000 Japanese patients with cancer. These mutators were designated as POLE category in the current study, and were considered according to the generation of biased substitutions such as C > A high and C > G low^8^. In addition, a high frequency of C > A with accompanying C > G has been observed in the common hypermutator tumour type derived from the lung, indicating a signature of tobacco exposure^23^. Although the characteristics of the POLE category were consistent with those of previous reports, our analysis revealed that this category was characterised by not only the staggering number of mutations but also by early onset colorectal or uterine tumour in comparison with the common hypermutator tumours. A similar finding was also observed in non-Japanese patients^24^. Furthermore, Shlien *et al*. recently reported that a patient with an inherited biallelic mismatch repair deficiency developed early onset brain tumours harbouring somatic POLE mutations^25^. Therefore, POLE-category status in the current study may be influenced by germline mutational status. Germline mutation should be also checked and validated in further studies.

Sporadic MSI usually arises from epigenetic silencing of the *MLH1* promoter via a global increase in CpG island methylation^26,27^, which has been correlated to down-regulation of its gene^28^. We checked MSI in POLE-category tumours using *MLH1* expression in GEP and by PCR of microsatellite markers, demonstrating loss of *MLH1* silencing and MSI negative status, respectively. Furthermore, although typical MSI is associated with somatic *BRAF* p.V600E status^29^, this mutation was not found in the POLE category. Previously, it has been demonstrated that an endometrial or colorectal cancer harbouring POLE mutations without MSI induces immune-suppression through inhibition of the PD1/PD-L1 axis^30,31^. Therefore, although MSI testing has attracted attention as a biomarker for PD-1 blockade^32^, an assessment of somatic mutation frequency and POLE mutation is instead recommended at least for patients with colorectal or uterine cancer.

We identified four genes (*SACS*, *LRP2*, *WDR87*, and *XIRP2*) as being highly susceptible to the POLE mutant in our analysis. Although several biased mutations were observed in these genes, a diverse profile pattern appeared. For example, TCT > TAT was found to be accumulated in *WDR87* in both this study and in a public repository. This propensity implies that susceptibility to POLE mutant status depended on the individual gene. To evaluate this susceptibility variation, we scored mutation propensity. The propensity mutations in the common hypermutator tumours rarely occurred without the aberrant *POLE*. Thus, propensity mutations may occur independently of POLE deficiency in POLE category tumours. To bypass this possibility, the frequency of these were thus subtracted as background. Our analysis identified not only two known three-letter base motifs (TCT and TCG) that accumulate massive quantities of mutation but also highly-mutated pattern (TTT) in the POLE category. Furthermore, T > G substitution was increased in comparison with the common hypermutator tumours, over 40% of which had occurred in the core of the TTT motif. However, T > G substitution is known to be associated with an imbalance of the dNTP pool^33,34^. Therefore, to determine the genes susceptible to POLE mutant status, mutation patterns of TCT > TAT and TCG > TTG except for TTT > TGT were utilised in the calculation of PS as the propensity mutation.

The propensity mutation patterns (TCT > TAT and TCG > TTG) were continually generated during defective POLE, implying inclusion of new (later-occurring) mutations. In these mutation patterns in 414 genes that were enriched in POLE-category, VAF score was decreased in comparison with other patterns. This finding may indicate that propensity mutation patterns include abundant mutations occurring subsequently.

We determined PS as a variation-susceptible indicator using the frequency of mutation pattern. This score was expected to represent a normal distribution when almost mutations randomly occurred in specific sequences (TCT and TCG) owing to defective POLE. However, the score of each gene that was enriched in the POLE category does not follow a normal distribution; additionally, IP was found on the Q-Q plot. This suggested that the subpopulation of low PS was less than the number of IP-enriched genes that had mutated independently of the POLE mutant. To clearly separate these genes, the indefinite period (CI of IP) was established as a border for buffering. Consequently, 414 genes that were enriched in the POLE category were classified into three periods. It is expected that the genes that mutated after the occurrence of the aberrant *POLE* would be concentrated in the POLE-dependent period (≥upper-CI), whereas the POLE-independent (<lower-CI) period was dominated by genes that mutated prior to or contemporary with this event, although this period also contained a certain number of genes unsusceptible to defective proofreading by POLE. Our classification was thus considered a reflection of the sequential shift of mutation profile resulting from the propensity of the POLE mutant. Accordingly, the *POLE* gene itself was classified in the POLE-independent period. Notably, a high mutation rate per protein-length of *PTEN* was observed in the indefinite period and the POLE-independent period using our and ICGC data sets, respectively, inferring that this gene was preferentially mutated during the early generation of the *POLE* mutation. Other cancer-related genes including *TSG* were also enriched in the POLE-dependent period. Therefore, we concluded that, based on the two-hit hypothesis, the somatic mutation of *POLE* and *PTEN* served as a primary trigger for tumourigenesis in POLE-category tumours.

Recently, characterisation of tumours has focused on the relationship between driver mutation and mutational signature. Poulos *et al*. revealed that mutations of methylated CpG dinucleotides were accumulated as potential driver events in POLE-mutant colorectal cancer^35^. In a pre-print repository (bioRxiv), Temko *et al*. reported that mutational signatures were affected by specific mutations in *APOBEC* and DNA mismatch repair genes^36^. Tumour-specific mutation pattern is considered to occur under conditions of defective DNA repair/replication-related genes. In our analysis, biased mutations in POLE mutants were scored based on the characteristic of visualisation of mutation pattern transition, resulting in the identification of *PTEN* mutation as potential tumourigenesis event that coincided roughly with the occurrence of defective *POLE*. However, significant accumulation of APC mutations that was detected in previous studies^35,36^ was not detected in our analysis. Quantification of mutation pattern in tumours with deficient DNA repair activity may constitute a helpful tool to estimate the procession of tumourigenesis.

*POLE* or *POLD1* mutation with the accompanying defect of DNA proofreading leads to reduction of fidelity in DNA replication, and a high level of somatic mutations in double-strand break repair (DSBR) genes (including HR and NHEJ) are also observed in these tissues^37^. However, it is unclear whether such mutants reduce activity of DSBR towards the excess accumulation of mutations. Our analysis revealed that HR repair activity based on gene expression was increased in POLE-category tumours despite the occurrence of *PTEN* mutations as an early event in tumourigenesis. As HR-related gene mutation is considered a compensatory event following PTEN-deficiency^22^, these findings imply that HR repair activity was sequentially shifted together with mutation accumulation after the occurrence of aberrant *PTEN*. Although POLE category tumours predominantly indicated the presence of Signature.10 based on deconstructSigs^14^, Signature.3 representing double-strand breaks was rare in these tumours (data not shown), suggesting that HR function may be gradually restored after occurrence of *PTEN* mutation. In particular, PTEN mutations initially compromise HR function, and then an increase of mutated DSBR genes that are generated by aberrant POLE may lead to a reversal of HR deficiency. To our knowledge, this study is the first report of a tumour exome profile that could be used to infer a footprint of somatic mutations.

Our classification showed that the sequential shift of mutation profile in genes had occurred in POLE-category tumours owing to the propensity of the POLE mutant effect. Similarly, to estimate the transition of mutation profile in pathways, this classification was applied to pathway analysis. *ASCC3* and *HIF1A* were categorised as POLE-independent, whereas the remaining POLE-category specific genes were susceptible to POLE mutant effects. Fms related tyrosine kinase 1 (*FLT1*) and kinase insert domain receptor (*KDR*), which are transcriptionally regulated by HIF1A, are known to function as VEGF receptors, affecting HIF1A activity through an autocrine loop^38,39^. In addition, ceruloplasmin (CP) stabilises HIF1A in a self-stimulating feedback loop^40,41^. Notably, HIF1A activity appears to be suppressed in POLE-category tumours. Activation of HIF1A under hypoxia leads to a decrease of HR function^19,42^; therefore, POLE-category tumours may compromise HIF1A activity via somatic mutations in itself and related genes to maintain the function of HR repair.

This study of the molecular and genomic mutational basis of hypermutator tumours harbouring POLE mutations allowed us to describe mutation footprints that could be classified according to POLE-dependent/-independent and indefinite periods. Notably, these periods illustrated the sequential shift of mutation that occurred via the defective proofreading of POLE in a clinical sample, identifying candidate genes as primary triggers of tumourigenesis or the suggested restoration of HR function resulting from mutation accumulation. The classification method developed through this study could be applied to tumours harbouring somatic mutations that were biased by defects of DNA repair.

In conclusion, the present study identified hypermutator tumours harbouring POLE mutation from among over 2,000 Japanese patients with cancer, and showed the similarity of observed molecular profile to that of previous studies. Although the aberrant POLE led to a mutation bias in the tumour, variation-insusceptible genes were also identified. Based on these characteristics, the transition of mutation in a clinical sample was classified into three periods. Furthermore, we revealed that the somatic mutation of *POLE* and *PTEN* represented a primary trigger for tumourigenesis in tumours with defective POLE proofreading ability. This is, to our knowledge, the first description of the two-hit hypothesis estimated from the classification of sequential mutation shift using a large-scale genomic study. A tracing of somatic mutation propensity may therefore provide new insight into the understanding of mutation-driven tumourigenesis.

## Methods

### Patients and specimens

Informed consent was obtained from all patients and the Institutional Review Board of Shizuoka Cancer Center approved all aspects of this study (Authorization Number: 25–33). All experiments using clinical samples were performed in accordance with the approved guidelines.

Each tumour and its surrounding normal tissue (≥0.1 g) were dissected from surgical specimens immediately after resection of the lesion. The tumour sample was visually assessed by a clinical pathologist in our hospital when tumour content was ≥50%. In addition, peripheral blood was collected as a control for WES.

### DNA and RNA isolation

For DNA analysis, tumour and normal tissues were immediately frozen in liquid nitrogen prior to DNA extraction. DNA was extracted from tissue and peripheral blood samples using a QIAamp DNA blood Mini Kit (Qiagen, Venlo, The Netherland). Purified DNA was quantified using a NanoDrop and Qubit 2.0 Fluorometer (Thermo Fisher Scientific, Waltham, MA, USA).

For RNA analysis, samples were immersed in RNAlater solution (Thermo Fisher Scientific), then stored overnight at 4 °C prior to RNA extraction. Total RNA was extracted from approximately 10 mg hashed tissue sample using the miRNeasy Mini Kit (Qiagen) according to manufacturer’s instruction. Initially, QIAzol reagent was applied to the tissue sample and then shaken with a 5-mm zirconia bead using a TissueLyser II (Qiagen) for 10 min at room temperature. The extracted RNA sample was quantified using a NanoDrop and its quality was checked using an Agilent 2100 Bioanalyzer (Agilent Technologies, Santa Clara, CA, USA). In our analysis, samples with RNA integrity number (RIN)^43^ <6.0 were discarded.

### Next-generation sequencing

The exome library for WES was constructed using an Ion Torrent AmpliSeq RDY Exome Kit (Thermo Fisher Scientific) in accordance with manufacturer recommended protocol^44,45^. For the construction, 100 ng DNA was used in the target amplification under the following conditions: 99 °C for 2 min, followed by 10 cycles at 95 °C for 15 s and 60 °C for 16 min, and a final hold at 10 °C. The amplicons were ligated with Ion Torrent Proton adapters (Thermo Fisher Scientific) at 22 °C for 30 min followed by 72 °C for 10 min, and the library was purified using Agencourt Ampure XT beads (Beckman Coulter). This exome library supplied 292,903 amplicons covering 57.7 Mb of the human genome comprising 34.8 Mb exons of 18,835 genes registered in RefSeq. The constructed library was quantified using quantitative PCR, and DNA was sequenced using a semiconductor DNA sequencer (Ion Torrent Proton Sequencer, Thermo Fisher Scientific) according to manufacturer’s instruction.

### Sequencing workflow for identification of somatic mutations

The semiconductor DNA sequencer-derived binary raw data were converted, using Torrent Suite software (ver. 4.4, Thermo Fisher Scientific), into sequence reads that were mapped to the reference human genome (UCSC hg19). At this step, sequence data derived from tumour and blood samples were individually analysed, and mapping results were saved as BAM files. Two BAM files were uploaded to the Ion Reporter system and analysed concurrently using AmpliSeq exome tumour-normal pair workflow (ver. 4.4, Thermo Fisher Scientific) with a Custom Hotspot file that specifies somatic and pathogenic mutations registered in COSMIC and ClinVar, respectively. The list of identified mutations was processed by in-house scripts to remove false positive calls including sequencer-derived error. Mutations fulfilling at least one of the following criteria were discarded as false positive: (1) quality score < 60, (2) depth of coverage < 20, (3) variant read observed in one strand only, (4) clipped sequence length < 100 (avg_clipped_length < 100), (5) variant located on either sequence end (avg_pos_as_fraction < 0.05), or (6) mutation matches to in-house false positive list. Parameters specified in criteria (4) and (5) were calculated by bam-readcount with option “-q 1” (ver. 0.8.0) (https://github.com/genome/bam-readcount). Our analysis focused on nonsynonymous SNVs located in an exon or splice site, and the mutation frequency on genome was then evaluated using 34.8 Mb exons. Arbitrary somatic mutations were manually inspected using the Integrative Genomics Viewer^46^ to avoid sequencer and amplicon-derived errors. Briefly, frequently mutated sites and variations that were barely detected in both tumour and blood were extracted from sequencing data. These somatic mutation candidates containing multiple nucleotide variation (~1,000 sites) were validated by Sanger sequencing to exclude false positive variation. The mutations that were not detected in Sanger sequencing were included in the in-house false positive list. To predict the effects of mutations, SnpEff^47^ and RefSeq were adopted as the source of curated and annotated sequences. Somatic mutations on the exome were annotated using the databases as follows: COSMIC^48^, ClinVar^49^, dbSNP^50^, UniProt^51^, and DrugBank^52^.

### Microarray analysis

Purified total RNA for GEP was amplified and fluorescent-labelled using a One-Color Low Input Quick Amp Labeling Kit (Agilent Technologies) according to manufacturer’s instruction. Hybridisation and scanning were performed as in a previous report^53,54^. Cy3-labelled cRNAs were hybridised to a SurePrint G3 Human Gene Expression 8 × 60 K v2 Microarray (Agilent Technologies), which has 50,599 probes capable of detecting 29,833 genes registered in the Entrez Gene Database. Fluorescent signal after hybridisation and washing was scanned using a DNA Microarray Scanner (Agilent Technologies), and then assessed by Agilent Feature Extraction software.

### Gene expression profiling (GEP)

To establish a correspondence between somatic mutation and gene expression, probes on the microarray were selected according to the reference human genome (UCSC hg19). Raw signal intensity derived from the scanned image was filtered by Agilent Flag Values to maintain reliability of microarray data, and then log-transformed and normalised to the 75th percentile. To compare gene expression between samples, z-score of the target gene was calculated from fold change (tumour vs normal in the same patient). In cases where tumour type created difficulty with respect to dissection of the normal sample, this expression profile was excluded from the analysis. These data were prepared and output using GeneSpring GX software (Agilent Technologies) and a Subio Platform. HR and NHEJ signatures were composed of multiple genes that showed up-regulated activation of these functions in a previous report^19^, and the signature score was calculated based on gene signature analysis using average of gene expression^28^. Microarray analysis was performed in accordance with MIAME guidelines^55^.

### Calculation of PS for classification

To discriminate whether a somatically mutated gene was susceptible to POLE mutant effects, a PS was employed as a variation-susceptible indicator. The PS was composed of frequencies of specific mutation patterns in POLE-category and common hypermutator tumours (classification described in Fig. 1b), and was calculated using the following formula:

1 $$PS=FM{P}_{POLEcategory}-FM{P}_{Commonhypermutator},$$

where FMP is the frequency of a mutation pattern in an individual gene. Then,

2 $$FMP( \% )=propensity\,mutation/total\,mutation\times 100,$$

where propensity mutation is the sum of TCT > TAT and TCG > TTG substitutions in a gene sequence. The above propensity mutation was exclusive of TTT > TGT. Total mutation is the number of all substitutions in each group (POLE category or common hypermutator).

An IP was determined using the Pearson correlation coefficient in the Q-Q plot of PS, and CI of IP (see Statistical analysis) was set as an indefinite region to clearly discriminate between POLE mutant-susceptible and unsusceptible genes.

### Pathway analysis

To estimate characteristic pathways, knowledge database analysis was performed based on sequential somatic mutation shift. Here, the transition of specific mutation pattern derived by defective protein (such as mutated POLE) was represented as sequential (somatic) mutation shift. To visualise the above shift, mutated genes in POLE category and common hypermutator were classified to three groups using PS (see Results section). For pathway analysis, our WES data and public data set of ICGC (https://dcc.icgc.org/) were prepared. Colon adenocarcinoma and uterine endometrium carcinoma data set obtained from ICGC (COAD-US and UCEC-US) were grouped into the POLE category and common hypermutator. POLE-category-specific mutations were then extracted, and their PS was calculated from this data set. The pathway analyses were performed using KeyMolnet software (ver.6.0.16.329, purchased from KM Data, http://www.immd.co.jp/en/index.html), which contains a stand-alone knowledge database. Upon interrelationship search of mutated genes in POLE category, the database was used with the following parameters: network termination, molecules; maximum number of path, 1; source, core and secondary; usage, positive/negative expression and direct relation. These analyses using knowledge database were scored by an internal index (H score), the elevated value of which indicated a high-relationship pathway.

### Statistical analysis

A significance of association of the mutations between the two groups was analysed using Fisher’s exact test. Microarray-derived gene expression data were normalised, and a significant difference in expression including the signature of gene set was calculated by Welch’s *t*-test. For mutation frequency and tumour cellularity as determined using PurBayes^56^, the assumptions of normality and the equality of two variances were tested by the Shapiro-Wilk test and *F*-test, respectively. Welch’s *t*-test was performed in the assumed normal distribution. Against comparison of samples assuming non-normal distribution, a Mann-Whitney-Wilcoxon or Brunner-Munzel test (also known as the generalised Wilcoxon test) was performed depending on the assumption of the *F*-test. To identify genes harbouring POLE-category-specific mutations, a Fisher’s exact test and the Benjamini-Hochberg procedure (*q* < 0.05) were carried out. Here, *P*-values < 0.05 were considered as significant. The break-point of segmented regression was estimated by Davies’ test^57,58^ (using R package ‘segmented’). In segmented regression, bootstrapping was employed to construct 99.999% CI, which was designated along with its underestimation by this method.

### Data availability

The WES data referenced during the study are available in a public repository that is accessible through the COSMIC and ICGC (https://dcc.icgc.org/) websites. The identifiers of individual samples are listed in Supplementary Table S2. The gene expression data including the signatures are described in Supplementary Table S3. The authors declare that all the other data supporting the findings of this study are available within the article and its supplementary information files and from the corresponding author upon reasonable request. The somatic mutation data of POLE-mutated samples and hypermutators from exome sequencing are available in the National Bioscience Database Center (NBDC) and Japanese Genotype-phenotype Archive (JGA) databases under the accession number hum0127 and JGAS00000000130, respectively.

## Electronic supplementary material

Supplementary information

Supplementary Table S3
