# Supplementary material for: Molecular profiling and sequential somatic mutation shift in hypermutator tumours harbouring POLE mutations
Source: Sci Rep. 2018 Jun 7;8:8700. doi: 10.1038/s41598-018-26967-4 (PMC5992218; doi:10.1038/s41598-018-26967-4)
Supplement: Supplementary file 1 — Supplementary information [file 41598_2018_26967_MOESM1_ESM.docx]

**Molecular profiling and sequential somatic mutation shift in hypermutator tumours harbouring POLE mutations**

Keiichi Hatakeyama^1,*^, Keiichi Ohshima^1^, Takeshi Nagashima^2,3^, Shumpei Ohnami^2^, Sumiko Ohnami^2^, Masakuni Serizawa^4^, Yuji Shimoda^2,3^, Koji Maruyama^5^, Yasuto Akiyama^6^, Kenichi Urakami^2^, Masatoshi Kusuhara^7^, Tohru Mochizuki^1^ & Ken Yamaguchi^8^

^1^Medical Genetics Division, Shizuoka Cancer Center Research Institute, Sunto-gun, Shizuoka 411-8777 Japan.

^2^Cancer Diagnostics Research Division, Shizuoka Cancer Center Research Institute, Sunto-gun, Shizuoka 411-8777 Japan.

^3^SRL Inc., Shinjuku-ku, Tokyo 163-0409 Japan.

^4^Drug Discovery and Development Division, Shizuoka Cancer Center Research Institute, Sunto-gun, Shizuoka 411-8777 Japan.

^5^Experimental Animal Facility, Shizuoka Cancer Center Research Institute, Sunto-gun, Shizuoka 411-8777 Japan.

^6^Immunotheraphy Division, Shizuoka Cancer Center Research Institute, Sunto-gun, Shizuoka 411-8777 Japan.

^7^Regional Resource Division, Shizuoka Cancer Center Research Institute, Sunto-gun, Shizuoka 411-8777 Japan.

^8^Shizuoka Cancer Center, Sunto-gun, Shizuoka 411-8777 Japan.

* Correspondence and requests for materials should be addressed to K.H. (email: k.hatakeyama@scchr.jp)


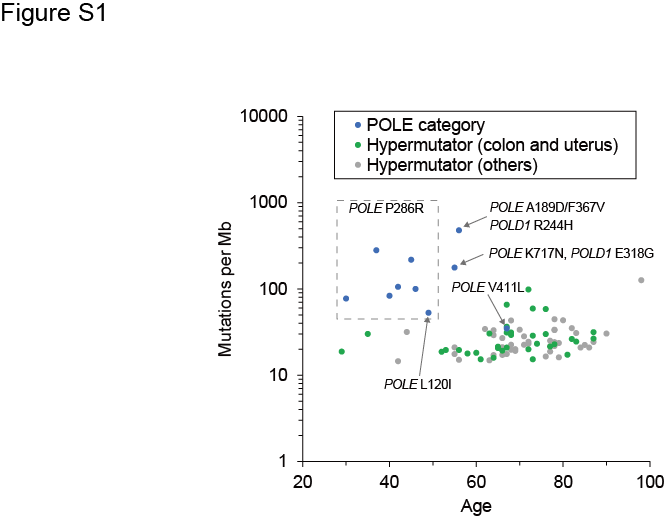


**Supplementary Figure S1.** **Frequency of somatic mutation as a function of age.** The substitution sites of *POLE* and *POLD1* in POLE-category tumours are represented in the graph. Significant difference between POLE-category and two hypermutator groups was observed for age (*p* value < 0.001).

**Supplementary Figure S2.** **Distribution of propensity mutation and sequence pattern in four genes susceptible to POLE mutant status, and inclusive of *POLE* and *PTEN* genes. a,** Distribution of all patterns of three-letter bases (bar chart) and frequency of mutation patterns in POLE-category tumours (line plot). The left- and right-pointed black arrows indicate corresponding y-axes of the bar chart and line plot, respectively. The red arrows indicate mutation motifs (TCG, TCT, and TTT) that are highly mutated by the POLE mutant. **b**, Number of mutations in the sequence motifs that are specifically mutated in POLE-category tumours. TCG>TTG, TCT>TAT, and TTT>TGT were counted as biased mutations in this bar chart.

**Supplementary Figure S3.** **Distribution of the biased mutation pattern in six genes (*SACS*, *WDR87*, *LRP2*, *XIRP2*, *POLE*, and *PTEN*) in the COSMIC database (http://cancer.sanger.ac.uk/cosmic). a,** The samples harbouring *POLE* p.P286R/p.V411L mutation were extracted from the database. LI, large intestine; EM, endometrium; BR, breast; ST, stomach. **b**, Sum of mutation patterns for each gene as obtained in our data set (left) and from COSMIC (right).

**Supplementary Figure S4.** **Flowchart of classification using propensity score (PS).** Cancer-related genes are extracted from mutated genes in all samples. These genes are separated into POLE-independent and -dependent periods by PS after extraction of POLE-category-specific genes using the Fisher's exact test and the Benjamini-Hochberg procedure (*q* < 0.05). The cancer-related genes that possess a low number of SNVs in the POLE-category group (SNV < 10) are discarded to enrich high-frequency mutated genes. After isolation using inflection point (IP), 99.999% confidence interval (CI) of IP was classified as the indefinite period.

**Supplementary Figure S5.** **Variant allele frequency (VAF) of propensity mutation patterns (TCT>TAT and TCG>TTG).** In the 414 genes identified in Supplementary Fig. S3, VAF of individual mutation patterns in each gene was calculated. Significant difference between propensity mutation patterns and others was observed in VAF. ***, *p* < 0.001.

**Supplementary Figure S6.** **Influence of inherent gene sequence and microsatellite instability (MSI) status on propensity score (PS).** **a**, Correlation of propensity score and appearance frequency of propensity sequences (TCT and TCG) in 414 genes that were enriched in POLE-category. **b**, Number and frequency of mutation patterns in other hypermutators with MSI-high/low and non-hypermutator tumours with MSI-high. **c**, Correlation analysis of FMPs. Correlation coefficients are represented in boxes. Symbol ‘X’ indicated as not significant. Tumours harbouring predominant Signature.6 (> 0.5 calculated using deconstructSigs^1^) are defined as MSI high, and the remaining samples are defined as MSI low.

**Supplementary Figure S7.** **Classification of POLE-category-specific mutations into POLE-independent and -dependent mutations using the ICGC data set. a**, Frequency density (left) and Q-Q plot (right) of propensity score (PS) in the genes harbouring POLE-category-specific mutations. Gene symbols correspond to Fig. 2c. The genes in the grey rectangle (confidence interval of IP) are classified in the group of indefinite mutation as a border for buffering. **b**, Comparison of the mutation rates for protein coding regions between cancer-related genes in the classified groups. Gene symbols are represented when more than 80 % of mutations could be classified as POLE category. Tumour suppressor genes and oncogene are shown with blue and red symbols, respectively.

**Supplementary Figure S8. Influences of microsatellite instability (MSI) status on homologous recombination (HR)-/non-homologous end joining (NHEJ)- signatures (a) and these mutations (b) in common hypermutators with or without MSI high.** Tumours harbouring predominant Signature.6 (> 0.5 calculated using deconstructSigs^1^) are defined as MSI high, and the remaining samples are defined as MSI low. Influences of tumour distribution on HR-/NHEJ-signatures (**c**) and these mutations (**d**) in POLE-category and common hypermutator tumours. Frequency of SNV represents proportion of mutation to HR- or NHEJ-related genes. No significant difference was observed between colon and uterine tumours. n.s., not significant.

**Supplementary Figure S9.** **Expression profiles of homologous recombination (HR)-related genes.** Gene signature analysis of HR genes (**a**) and gene expression of *TTK* and *DEPDC1* (**c**) between samples with/without *PTEN* mutation in colon and uterine tumours including non-hypermutator. **, *p* < 0.01; ***, *p* < 0.001.


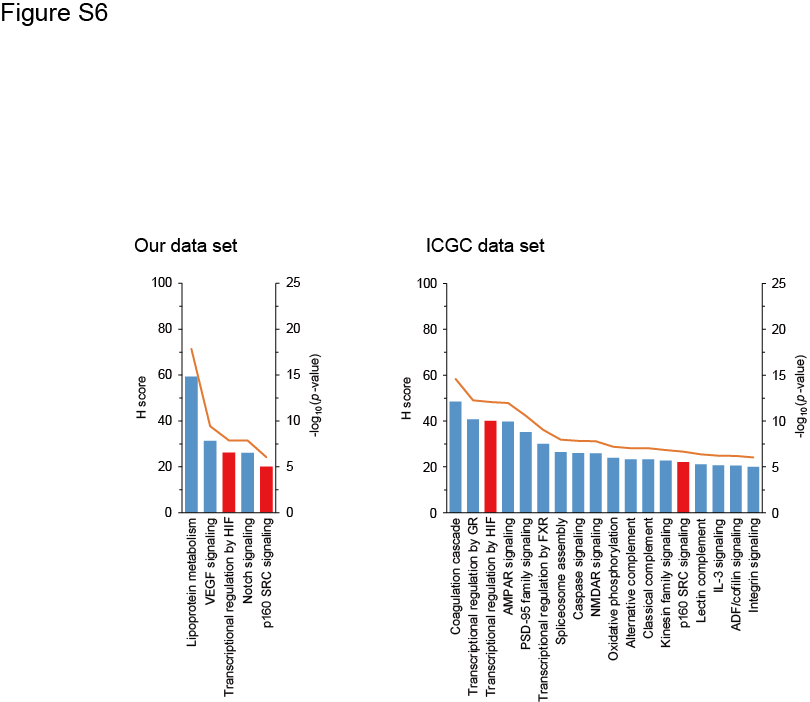


**Supplementary Figure S10.** **Pathway profiles of POLE-category tumours in ICGC (right) and our (left) data sets.** Individual pathways are shown based on an interrelationship search of genes that were harboured by individuals with POLE-category tumours. Red bars indicate pathways shared in both data sets.

**Supplementary Figure S11.** **Pathway alterations in POLE-category tumours using the ICGC data set. a**, The p160 steroid receptor co-activator (SRC) signalling and transcriptional regulation (TR) by hypoxia inducible factor (HIF) pathways are altered through accumulation of somatic mutations. Alteration frequencies are expressed as a percentage of all cases. The genes that were significantly mutated between our study and public database are represented. **b**, Mutation patterns in the p160 SRC signalling and TR by HIF pathways.

**Supplementary Table S1. List of 1178 genes harbouring POLE-category specific mutations**

| Gene symbol | Number of SNVs | | *q*-value | PS | cancer-related gene |
| --- | --- | --- | --- | --- | --- |
|  | POLE category | Common hypermutator |  |  |  |
| *ADGRG4* | 27 | 1 | 0.0000847 | 29.63 |  |
| *ZNF99* | 23 | 2 | 0.000254 | -2.17 |  |
| *AKAP12* | 19 | 0 | 0.000375 | 52.63 |  |
| *CFAP70* | 16 | 1 | 0.000375 | 56.25 |  |
| *LRRIQ1* | 23 | 1 | 0.000375 | 60.87 |  |
| *NALCN* | 22 | 1 | 0.000375 | 31.82 |  |
| *RIMS2* | 17 | 1 | 0.000375 | 52.94 |  |
| *SLC39A10* | 10 | 0 | 0.000485 | 10.00 |  |
| *TRPM6* | 18 | 0 | 0.000485 | 50.00 |  |
| *ZNF718* | 12 | 0 | 0.000485 | 58.33 |  |
| *GOLGB1* | 38 | 5 | 0.000701 | 55.26 |  |
| *FAM208A* | 15 | 1 | 0.001082 | 20.00 |  |
| *RAD51AP2* | 18 | 2 | 0.001082 | 61.11 |  |
| *POLE* | 12 | 5 | 0.001273 | 8.33 | yes |
| *WDR87* | 41 | 6 | 0.001273 | 29.67 |  |
| *ALS2CR11* | 20 | 1 | 0.001273 | 45.00 |  |
| *ARAP2* | 12 | 1 | 0.001273 | 66.67 |  |
| *EML5* | 14 | 1 | 0.001273 | 28.57 |  |
| *F8* | 16 | 1 | 0.001273 | 37.50 |  |
| *FGD6* | 10 | 1 | 0.001273 | 60.00 |  |
| *FILIP1* | 14 | 1 | 0.001273 | 50.00 |  |
| *IQGAP2* | 10 | 2 | 0.001273 | 40.00 |  |
| *ITIH2* | 16 | 1 | 0.001273 | 50.00 |  |
| *PLCZ1* | 13 | 1 | 0.001273 | 30.77 |  |
| *SPATA31D1* | 22 | 1 | 0.001273 | 59.09 |  |
| *TMTC1* | 13 | 1 | 0.001273 | 23.08 |  |
| *WDR17* | 11 | 1 | 0.001273 | 63.64 |  |
| *ZNF644* | 15 | 1 | 0.001273 | 53.33 |  |
| *ZNF678* | 13 | 1 | 0.001273 | -23.08 |  |
| *ABCC12* | 16 | 0 | 0.001273 | 56.25 |  |
| *DCT* | 10 | 0 | 0.001273 | 60.00 |  |
| *DSG1* | 11 | 0 | 0.001273 | 45.45 |  |
| *EXOC6B* | 12 | 0 | 0.001273 | 25.00 |  |
| *LCA5* | 11 | 0 | 0.001273 | 72.73 |  |
| *RAD54B* | 11 | 0 | 0.001273 | 54.55 |  |
| *RBM44* | 14 | 0 | 0.001273 | 28.57 |  |
| *TTC3* | 12 | 0 | 0.001273 | 41.67 |  |
| *ZNF564* | 11 | 0 | 0.001273 | 54.55 |  |
| *ZNF737* | 12 | 0 | 0.001273 | 25.00 |  |
| *EYS* | 39 | 5 | 0.001285 | 26.15 |  |
| *CMYA5* | 40 | 8 | 0.002776 | 22.50 |  |
| *ATP12A* | 16 | 4 | 0.002776 | 56.25 |  |
| *FGD5* | 12 | 4 | 0.002776 | 25.00 |  |
| *IGSF10* | 22 | 3 | 0.002776 | 36.36 |  |
| *KIF20B* | 22 | 4 | 0.002776 | 34.09 |  |
| *MMRN1* | 16 | 3 | 0.002776 | 43.75 |  |
| *SAMD9* | 22 | 4 | 0.002776 | 59.09 | yes |
| *TOPAZ1* | 28 | 6 | 0.002776 | 53.57 |  |
| *ANKRD17* | 12 | 2 | 0.002776 | 50.00 |  |
| *ANKRD50* | 14 | 2 | 0.002776 | 28.57 |  |
| *C5orf42* | 22 | 3 | 0.002776 | 31.82 |  |
| *CEP290* | 13 | 2 | 0.002776 | 46.15 |  |
| *COL4A6* | 16 | 2 | 0.002776 | 43.75 |  |
| *FAM186A* | 13 | 2 | 0.002776 | 46.15 |  |
| *HEATR6* | 12 | 2 | 0.002776 | 83.33 |  |
| *ITSN1* | 12 | 4 | 0.002776 | 16.67 |  |
| *KTN1* | 10 | 2 | 0.002776 | -20.00 | yes |
| *LATS1* | 12 | 2 | 0.002776 | 50.00 | yes |
| *MYH1* | 18 | 2 | 0.002776 | 61.11 |  |
| *OTOA* | 12 | 2 | 0.002776 | -16.67 |  |
| *PLCG2* | 19 | 2 | 0.002776 | 31.58 | yes |
| *SYCP2* | 22 | 2 | 0.002776 | 40.91 |  |
| *ZDBF2* | 19 | 2 | 0.002776 | 36.84 |  |
| *FSIP2* | 64 | 9 | 0.004004 | 39.06 |  |
| *VCAN* | 47 | 9 | 0.004004 | 25.53 |  |
| *ZFHX4* | 37 | 10 | 0.004004 | 35.14 |  |
| *PIEZO2* | 23 | 7 | 0.004004 | 43.48 |  |
| *PTEN* | 17 | 4 | 0.004004 | 23.53 | yes (TSG) |
| *PTPRZ1* | 23 | 5 | 0.004004 | 21.74 |  |
| *SCN3A* | 13 | 5 | 0.004004 | 10.77 |  |
| *ARHGAP11A* | 10 | 1 | 0.004004 | 50.00 |  |
| *ARID4A* | 11 | 1 | 0.004004 | 45.45 |  |
| *CAGE1* | 12 | 1 | 0.004004 | -58.33 |  |
| *CDH19* | 12 | 1 | 0.004004 | 50.00 |  |
| *FAM171B* | 12 | 1 | 0.004004 | 50.00 |  |
| *IQCH* | 11 | 1 | 0.004004 | 54.55 |  |
| *LY75* | 11 | 1 | 0.004004 | 63.64 | yes |
| *MED13* | 13 | 1 | 0.004004 | -53.85 |  |
| *PIK3C2G* | 12 | 1 | 0.004004 | 33.33 | yes |
| *PZP* | 13 | 1 | 0.004004 | 53.85 |  |
| *SNX19* | 10 | 1 | 0.004004 | 30.00 |  |
| *STOX1* | 10 | 1 | 0.004004 | 30.00 |  |
| *SYCP1* | 17 | 1 | 0.004004 | 52.94 |  |
| *TRPM7* | 10 | 1 | 0.004004 | 40.00 |  |
| *USPL1* | 14 | 1 | 0.004004 | -71.43 |  |
| *ZNF229* | 10 | 1 | 0.004004 | 10.00 |  |
| *CCDC150* | 11 | 0 | 0.004004 | 27.27 |  |
| *CFAP58* | 15 | 0 | 0.004004 | 46.67 |  |
| *L1TD1* | 10 | 0 | 0.004004 | 50.00 |  |
| *MYBPC1* | 10 | 0 | 0.004004 | 20.00 |  |
| *PCDHGB5* | 12 | 0 | 0.004004 | 25.00 |  |
| *TLR2* | 10 | 0 | 0.004004 | 50.00 |  |
| *TLR8* | 10 | 0 | 0.004004 | 40.00 |  |
| *TMEM131* | 11 | 0 | 0.004004 | 36.36 |  |
| *ZNF573* | 11 | 0 | 0.004004 | 45.45 |  |
| *ZNF841* | 13 | 0 | 0.004004 | 53.85 |  |
| *ADAMTS9* | 16 | 4 | 0.004392 | 37.50 |  |
| *ATG2B* | 15 | 4 | 0.004392 | 60.00 |  |
| *CENPF* | 28 | 4 | 0.004392 | 53.57 |  |
| *CHL1* | 18 | 3 | 0.004392 | -27.78 |  |
| *EFCAB5* | 11 | 3 | 0.004392 | 72.73 |  |
| *JAKMIP2* | 10 | 4 | 0.004392 | 15.00 |  |
| *KIAA1551* | 15 | 3 | 0.004392 | 6.67 |  |
| *MAGEC1* | 19 | 4 | 0.004392 | 42.11 |  |
| *MAP3K19* | 19 | 3 | 0.004392 | 19.30 |  |
| *SLC12A1* | 13 | 3 | 0.004392 | 38.46 |  |
| *SYNPO2* | 10 | 3 | 0.004392 | 50.00 |  |
| *TET1* | 14 | 2 | 0.004392 | 42.86 | yes |
| *ZNF292* | 23 | 4 | 0.004392 | 26.09 |  |
| *ZNF416* | 12 | 4 | 0.004392 | 33.33 |  |
| *CCDC168* | 89 | 12 | 0.004503 | 50.56 |  |
| *LRP1B* | 60 | 11 | 0.004503 | 25.91 | yes |
| *ATM* | 22 | 5 | 0.006958 | 16.36 | yes (TSG) |
| *CEP192* | 14 | 6 | 0.006958 | 42.86 |  |
| *CSMD3* | 35 | 12 | 0.006958 | 31.67 | yes |
| *DYSF* | 25 | 6 | 0.006958 | 39.33 |  |
| *SCN2A* | 15 | 6 | 0.006958 | 53.33 |  |
| *USP34* | 19 | 7 | 0.006958 | 4.51 |  |
| *APOB* | 50 | 13 | 0.00842 | 1.23 |  |
| *DNAH11* | 43 | 13 | 0.00842 | 38.82 |  |
| *LRP2* | 40 | 12 | 0.00842 | 41.67 | yes |
| *STARD9* | 31 | 11 | 0.00842 | 29.62 |  |
| *XIRP2* | 44 | 13 | 0.00842 | 53.67 |  |
| *ASH1L* | 15 | 4 | 0.008534 | 41.67 |  |
| *ATRX* | 14 | 4 | 0.008534 | 28.57 | yes (TSG) |
| *CACNA1D* | 14 | 4 | 0.008534 | 42.86 | yes |
| *CRB1* | 15 | 5 | 0.008534 | 20.00 |  |
| *DMXL1* | 14 | 5 | 0.008534 | 42.86 |  |
| *KCNT2* | 13 | 4 | 0.008534 | 38.46 |  |
| *LRRK2* | 28 | 4 | 0.008534 | 35.71 | yes |
| *MAP2* | 19 | 4 | 0.008534 | 36.84 |  |
| *MORC1* | 12 | 4 | 0.008534 | 50.00 |  |
| *MYO3B* | 18 | 3 | 0.008534 | 33.33 |  |
| *N4BP2* | 12 | 4 | 0.008534 | 50.00 |  |
| *PCNX1* | 12 | 4 | 0.008534 | 58.33 | yes |
| *PRUNE2* | 21 | 5 | 0.008534 | 52.38 |  |
| *REV3L* | 23 | 3 | 0.008534 | 1.45 |  |
| *SI* | 14 | 4 | 0.008534 | 42.86 |  |
| *SLC4A10* | 15 | 4 | 0.008534 | 60.00 |  |
| *SPHKAP* | 13 | 6 | 0.008534 | 46.15 |  |
| *TEX15* | 19 | 5 | 0.008534 | 36.84 |  |
| *TRPC6* | 10 | 4 | 0.008534 | 5.00 |  |
| *VPS13A* | 20 | 3 | 0.008534 | 26.67 |  |
| *ABCA5* | 14 | 2 | 0.008534 | 21.43 |  |
| *ABCA6* | 15 | 2 | 0.008534 | 60.00 |  |
| *ABCA9* | 11 | 2 | 0.008534 | 45.45 |  |
| *ANKRD12* | 15 | 2 | 0.008534 | 53.33 |  |
| *ARHGAP35* | 12 | 2 | 0.008534 | 41.67 | yes |
| *CATSPERB* | 10 | 2 | 0.008534 | 40.00 |  |
| *CDH2* | 12 | 1 | 0.008534 | 41.67 | yes |
| *EPRS* | 11 | 2 | 0.008534 | 27.27 |  |
| *TNNI3K* | 10 | 2 | 0.008534 | 30.00 | yes |
| *GABRG2* | 13 | 2 | 0.008534 | 30.77 |  |
| *GOLGA4* | 20 | 1 | 0.008534 | 65.00 |  |
| *GPR155* | 11 | 2 | 0.008534 | -13.64 |  |
| *IL1RAPL1* | 12 | 2 | 0.008534 | 50.00 |  |
| *LEPR* | 10 | 2 | 0.008534 | 10.00 |  |
| *MGAM* | 14 | 3 | 0.008534 | 35.71 |  |
| *MIA3* | 16 | 3 | 0.008534 | 31.25 |  |
| *NBEAL1* | 18 | 2 | 0.008534 | 50.00 |  |
| *NSD1* | 11 | 2 | 0.008534 | -4.55 | yes |
| *PEAK1* | 12 | 4 | 0.008534 | 41.67 |  |
| *PRTG* | 11 | 2 | 0.008534 | 36.36 |  |
| *THNSL1* | 10 | 2 | 0.008534 | 40.00 |  |
| *THSD7B* | 14 | 4 | 0.008534 | 42.86 |  |
| *TRAPPC8* | 12 | 2 | 0.008534 | 58.33 |  |
| *ZGRF1* | 12 | 3 | 0.008534 | 33.33 |  |
| *ZNF616* | 16 | 2 | 0.008534 | 75.00 |  |
| *MXRA5* | 28 | 13 | 0.010764 | 26.92 | yes |
| *COL12A1* | 23 | 7 | 0.010764 | 29.19 |  |
| *COL6A5* | 32 | 7 | 0.010764 | 40.63 |  |
| *DMD* | 35 | 7 | 0.010764 | 28.57 |  |
| *MYH2* | 19 | 8 | 0.010764 | 57.89 |  |
| *NAV3* | 14 | 9 | 0.010764 | 50.00 | yes |
| *VPS13B* | 17 | 9 | 0.010764 | 30.07 |  |
| *ABCC4* | 10 | 1 | 0.010764 | 50.00 | yes |
| *ADGRF5* | 11 | 1 | 0.010764 | 0.00 |  |
| *BDP1* | 16 | 1 | 0.010764 | 31.25 |  |
| *CP* | 11 | 1 | 0.010764 | 54.55 |  |
| *DENND4C* | 13 | 1 | 0.010764 | 30.77 |  |
| *ERC2* | 10 | 1 | 0.010764 | -50.00 |  |
| *HEMGN* | 13 | 1 | 0.010764 | -38.46 |  |
| *KCNU1* | 13 | 1 | 0.010764 | 15.38 |  |
| *KIR3DL2* | 10 | 1 | 0.010764 | 20.00 |  |
| *MAP3K13* | 12 | 1 | 0.010764 | 25.00 | yes |
| *SENP7* | 13 | 1 | 0.010764 | 53.85 |  |
| *SLC9C2* | 11 | 1 | 0.010764 | 63.64 |  |
| *TBC1D32* | 11 | 1 | 0.010764 | 72.73 |  |
| *TTC14* | 12 | 1 | 0.010764 | 50.00 |  |
| *UGGT1* | 11 | 1 | 0.010764 | 72.73 |  |
| *ZNF18* | 10 | 1 | 0.010764 | 50.00 |  |
| *ZNF596* | 10 | 1 | 0.010764 | 70.00 |  |
| *ZNF615* | 12 | 1 | 0.010764 | 58.33 |  |
| *CEMIP* | 10 | 0 | 0.010764 | 40.00 |  |
| *NCOA1* | 10 | 0 | 0.010764 | 30.00 | yes |
| *UGT2B15* | 10 | 0 | 0.010764 | 40.00 |  |
| *ZNF37A* | 10 | 0 | 0.010764 | 50.00 |  |
| *BTAF1* | 10 | 5 | 0.011226 | 0.00 |  |
| *CACNA2D3* | 12 | 5 | 0.011226 | 66.67 |  |
| *CEP295* | 18 | 5 | 0.011226 | 18.89 |  |
| *CFTR* | 16 | 5 | 0.011226 | 16.25 |  |
| *CHD7* | 12 | 5 | 0.011226 | -3.33 |  |
| *CPS1* | 14 | 4 | 0.011226 | 17.86 |  |
| *DUOX1* | 11 | 5 | 0.011226 | 36.36 |  |
| *EPHA3* | 14 | 6 | 0.011226 | 7.14 | yes |
| *ICE1* | 17 | 6 | 0.011226 | 29.41 |  |
| *SCN7A* | 16 | 5 | 0.011226 | 36.25 |  |
| *SCN9A* | 14 | 6 | 0.011226 | 35.71 | yes |
| *SPG11* | 17 | 5 | 0.011226 | 27.06 |  |
| *UNC13C* | 18 | 5 | 0.011226 | 44.44 |  |
| *URB2* | 10 | 5 | 0.011226 | 70.00 |  |
| *DNAH3* | 35 | 9 | 0.012162 | 34.29 |  |
| *DNAH6* | 28 | 9 | 0.012162 | 38.89 |  |
| *ADCY10* | 10 | 3 | 0.012318 | 40.00 |  |
| *ATAD5* | 13 | 3 | 0.012318 | 46.15 |  |
| *ATP8B4* | 12 | 3 | 0.012318 | 33.33 |  |
| *BCLAF1* | 13 | 3 | 0.012318 | 46.15 | yes |
| *CCDC141* | 12 | 3 | 0.012318 | 50.00 |  |
| *CEP152* | 11 | 3 | 0.012318 | 63.64 |  |
| *DOCK10* | 11 | 4 | 0.012318 | 63.64 |  |
| *DPP10* | 12 | 3 | 0.012318 | 33.33 |  |
| *EFHC1* | 10 | 3 | 0.012318 | 6.67 |  |
| *EML6* | 13 | 3 | 0.012318 | -10.26 |  |
| *EPHA6* | 14 | 4 | 0.012318 | -14.29 |  |
| *GALNT13* | 11 | 3 | 0.012318 | 54.55 |  |
| *KIAA2026* | 11 | 2 | 0.012318 | -13.64 |  |
| *LAMA3* | 15 | 4 | 0.012318 | 40.00 |  |
| *LRPPRC* | 11 | 4 | 0.012318 | 18.18 |  |
| *MYH8* | 23 | 3 | 0.012318 | 5.80 |  |
| *NUP210L* | 15 | 5 | 0.012318 | 26.67 |  |
| *PRDM9* | 12 | 4 | 0.012318 | 75.00 |  |
| *RLF* | 20 | 3 | 0.012318 | 55.00 |  |
| *RNF17* | 13 | 3 | 0.012318 | 30.77 |  |
| *SEMA6D* | 13 | 3 | 0.012318 | 30.77 |  |
| *SLFN14* | 13 | 3 | 0.012318 | 53.85 |  |
| *TRIP11* | 13 | 2 | 0.012318 | -69.23 | yes |
| *ZNF709* | 10 | 3 | 0.012318 | 56.67 |  |
| *ZNF782* | 12 | 3 | 0.012318 | 50.00 |  |
| *ANK3* | 27 | 13 | 0.021376 | 36.75 |  |
| *ADGRV1* | 39 | 12 | 0.021433 | 41.03 |  |
| *KMT2C* | 15 | 10 | 0.021433 | 43.33 | yes (TSG) |
| *PKD1L2* | 22 | 9 | 0.021433 | 22.73 |  |
| *SPEG* | 25 | 12 | 0.021433 | 28.00 |  |
| *ADGB* | 17 | 7 | 0.022172 | 32.77 |  |
| *AKAP6* | 25 | 6 | 0.022172 | 56.00 |  |
| *ARHGAP31* | 11 | 6 | 0.022172 | 37.88 |  |
| *GPR179* | 15 | 7 | 0.022172 | 4.76 |  |
| *NWD2* | 17 | 7 | 0.022172 | 52.94 |  |
| *FAT3* | 32 | 16 | 0.022867 | 37.50 | yes |
| *NEB* | 61 | 16 | 0.022867 | 34.73 |  |
| *PCLO* | 47 | 18 | 0.022867 | 38.30 |  |
| *SACS* | 43 | 21 | 0.022867 | 29.90 |  |
| *USH2A* | 45 | 16 | 0.022867 | 16.81 |  |
| *ABCA10* | 10 | 2 | 0.022867 | 30.00 |  |
| *ADAM29* | 10 | 2 | 0.022867 | 20.00 |  |
| *ANKRD26* | 11 | 2 | 0.022867 | 54.55 |  |
| *ANKRD30A* | 13 | 4 | 0.022867 | 38.46 |  |
| *ATP13A5* | 15 | 3 | 0.022867 | 26.67 |  |
| *C18orf63* | 10 | 1 | 0.022867 | 50.00 |  |
| *C9orf84* | 15 | 2 | 0.022867 | 60.00 |  |
| *CCDC7* | 11 | 2 | 0.022867 | 45.45 |  |
| *CDH9* | 11 | 2 | 0.022867 | 54.55 |  |
| *CFAP47* | 12 | 3 | 0.022867 | 41.67 |  |
| *CNKSR2* | 11 | 1 | 0.022867 | 27.27 |  |
| *CNTN6* | 19 | 2 | 0.022867 | 47.37 |  |
| *CORIN* | 16 | 2 | 0.022867 | 12.50 |  |
| *CPED1* | 12 | 2 | 0.022867 | 33.33 |  |
| *DPCR1* | 12 | 2 | 0.022867 | 58.33 |  |
| *ECT2L* | 10 | 2 | 0.022867 | 70.00 | yes |
| *EEA1* | 15 | 2 | 0.022867 | 53.33 |  |
| *EXPH5* | 11 | 2 | 0.022867 | 36.36 |  |
| *FMNL2* | 12 | 2 | 0.022867 | 33.33 |  |
| *FMNL3* | 10 | 2 | 0.022867 | 50.00 |  |
| *KIF14* | 13 | 2 | 0.022867 | 38.46 |  |
| *LRP12* | 11 | 2 | 0.022867 | -22.73 |  |
| *MBD5* | 10 | 2 | 0.022867 | 60.00 |  |
| *MYOM2* | 14 | 2 | 0.022867 | 71.43 |  |
| *PDE4D* | 11 | 2 | 0.022867 | 54.55 |  |
| *PHF3* | 16 | 2 | 0.022867 | 37.50 |  |
| *RASA1* | 16 | 2 | 0.022867 | 50.00 | yes |
| *SGIP1* | 10 | 2 | 0.022867 | 50.00 |  |
| *SGO2* | 14 | 2 | 0.022867 | 57.14 | yes |
| *TBC1D9* | 10 | 2 | 0.022867 | -10.00 |  |
| *TMEM63C* | 10 | 2 | 0.022867 | 0.00 |  |
| *TMPRSS15* | 11 | 2 | 0.022867 | 27.27 |  |
| *TRIM36* | 10 | 2 | 0.022867 | 70.00 |  |
| *TRPC4* | 10 | 2 | 0.022867 | 40.00 |  |
| *VCAM1* | 12 | 2 | 0.022867 | 66.67 |  |
| *VWA8* | 11 | 2 | 0.022867 | 63.64 |  |
| *ZNF208* | 10 | 2 | 0.022867 | 60.00 |  |
| *ZNF594* | 10 | 2 | 0.022867 | 40.00 |  |
| *ZNF721* | 13 | 1 | 0.022867 | 38.46 |  |
| *ZNF91* | 10 | 2 | 0.022867 | 80.00 |  |
| *ADNP2* | 10 | 3 | 0.023663 | 20.00 |  |
| *AKAP3* | 11 | 4 | 0.023663 | 63.64 |  |
| *ALPK2* | 10 | 5 | 0.023663 | 20.00 | yes |
| *ASCC3* | 14 | 5 | 0.023663 | -24.29 |  |
| *C2orf16* | 11 | 4 | 0.023663 | 45.45 |  |
| *C5* | 11 | 4 | 0.023663 | 38.64 |  |
| *CACNA2D1* | 10 | 4 | 0.023663 | 40.00 |  |
| *CAPRIN2* | 13 | 4 | 0.023663 | 13.46 |  |
| *DOCK8* | 12 | 5 | 0.023663 | 16.67 |  |
| *DUOX2* | 12 | 5 | 0.023663 | 58.33 |  |
| *FREM1* | 21 | 3 | 0.023663 | 28.57 |  |
| *GREB1L* | 10 | 4 | 0.023663 | 20.00 |  |
| *HIF1A* | 10 | 4 | 0.023663 | 5.00 | yes |
| *KAT2B* | 10 | 4 | 0.023663 | 50.00 |  |
| *KDR* | 13 | 4 | 0.023663 | 30.77 | yes |
| *KIAA0825* | 14 | 4 | 0.023663 | 57.14 |  |
| *KMT2A* | 14 | 5 | 0.023663 | 64.29 | yes |
| *MAP3K15* | 16 | 4 | 0.023663 | 37.50 | yes |
| *MMS22L* | 12 | 3 | 0.023663 | 50.00 |  |
| *MSH6* | 11 | 3 | 0.023663 | 63.64 | yes (TSG) |
| *NAA15* | 16 | 4 | 0.023663 | 56.25 |  |
| *NCOR1* | 15 | 3 | 0.023663 | 33.33 | yes (TSG) |
| *PCDH11X* | 10 | 4 | 0.023663 | 50.00 |  |
| *POSTN* | 10 | 4 | 0.023663 | 25.00 |  |
| *ROCK2* | 10 | 4 | 0.023663 | 10.00 |  |
| *ROS1* | 20 | 5 | 0.023663 | 45.00 | yes |
| *RSPH4A* | 10 | 2 | 0.023663 | 50.00 |  |
| *SLITRK6* | 10 | 4 | 0.023663 | 40.00 |  |
| *TICRR* | 14 | 4 | 0.023663 | 28.57 |  |
| *TMC3* | 10 | 3 | 0.023663 | -23.33 |  |
| *TNR* | 13 | 5 | 0.023663 | 23.08 |  |
| *TPR* | 20 | 4 | 0.023663 | 0.00 | yes |
| *ADAMTS5* | 14 | 10 | 0.027864 | 54.29 |  |
| *UNC79* | 23 | 9 | 0.027864 | 28.02 |  |
| *CACNA1E* | 14 | 7 | 0.027864 | 21.43 |  |
| *COL6A3* | 34 | 8 | 0.027864 | 41.18 |  |
| *COL6A6* | 21 | 7 | 0.027864 | 23.81 |  |
| *DCC* | 14 | 7 | 0.027864 | 42.86 | yes |
| *MYO9A* | 18 | 7 | 0.027864 | 50.00 |  |
| *NBEA* | 23 | 8 | 0.027864 | 47.83 |  |
| *SPEN* | 12 | 7 | 0.027864 | 41.67 | yes |
| *CCDC146* | 10 | 0 | 0.027864 | 20.00 |  |
| *CCDC170* | 10 | 1 | 0.027864 | 50.00 |  |
| *CD180* | 11 | 1 | 0.027864 | 45.45 |  |
| *MAGI2* | 10 | 1 | 0.027864 | 20.00 |  |
| *NLRC4* | 12 | 1 | 0.027864 | 25.00 |  |
| *NLRP7* | 11 | 1 | 0.027864 | 36.36 |  |
| *NUP133* | 10 | 1 | 0.027864 | 40.00 |  |
| *ROCK1* | 10 | 1 | 0.027864 | 70.00 |  |
| *SIX4* | 10 | 1 | 0.027864 | 10.00 |  |
| *SNTG1* | 12 | 1 | 0.027864 | 8.33 |  |
| *ST18* | 10 | 1 | 0.027864 | 30.00 |  |
| *TLR5* | 11 | 1 | 0.027864 | 54.55 |  |
| *TRHDE* | 14 | 1 | 0.027864 | 57.14 |  |
| *ZNF257* | 10 | 1 | 0.027864 | 80.00 |  |
| *ZNF442* | 12 | 1 | 0.027864 | 50.00 |  |
| *ZNF470* | 12 | 1 | 0.027864 | 66.67 |  |
| *ZNF676* | 11 | 1 | 0.027864 | 72.73 |  |
| *ZNF700* | 11 | 1 | 0.027864 | 36.36 |  |
| *ITIH6* | 13 | 0 | 0.027864 | 0.00 |  |
| *ARHGAP32* | 10 | 5 | 0.029543 | 70.00 |  |
| *BPTF* | 12 | 5 | 0.029543 | -15.00 |  |
| *CACNA1I* | 11 | 5 | 0.029543 | 27.27 |  |
| *CENPE* | 13 | 6 | 0.029543 | 44.87 |  |
| *EPHA5* | 14 | 5 | 0.029543 | 28.57 | yes |
| *FER1L6* | 18 | 7 | 0.029543 | 44.44 |  |
| *HEPHL1* | 13 | 5 | 0.029543 | 38.46 |  |
| *ITPR1* | 18 | 10 | 0.029543 | 33.33 |  |
| *KIAA1549L* | 17 | 6 | 0.029543 | 23.53 |  |
| *USF3* | 14 | 5 | 0.029543 | 50.00 | yes |
| *LCT* | 21 | 4 | 0.029543 | 38.10 |  |
| *MAP3K5* | 10 | 5 | 0.029543 | 20.00 |  |
| *MROH2B* | 13 | 5 | 0.029543 | 30.77 |  |
| *MYCBP2* | 17 | 8 | 0.029543 | 35.29 |  |
| *MYO5A* | 12 | 5 | 0.029543 | 25.00 | yes |
| *NAV2* | 14 | 5 | 0.029543 | 28.57 |  |
| *NOTCH2* | 11 | 5 | 0.029543 | 27.27 | yes (TSG) |
| *OTOGL* | 16 | 5 | 0.029543 | 36.25 |  |
| *TAF1L* | 19 | 5 | 0.029543 | 27.37 | yes |
| *TEP1* | 15 | 7 | 0.029543 | 25.71 |  |
| *TMEM132C* | 10 | 6 | 0.029543 | 20.00 |  |
| *TRPC5* | 10 | 5 | 0.029543 | 30.00 |  |
| *TSHZ3* | 10 | 5 | 0.029543 | 40.00 | yes |
| *ADGRB3* | 18 | 3 | 0.030514 | 61.11 | yes |
| *ANKRD31* | 16 | 4 | 0.030514 | 62.50 |  |
| *AQR* | 10 | 3 | 0.030514 | 70.00 |  |
| *ARHGEF11* | 11 | 3 | 0.030514 | 45.45 |  |
| *ATR* | 14 | 4 | 0.030514 | 57.14 | yes |
| *CBLB* | 12 | 3 | 0.030514 | 33.33 | yes |
| *CEP128* | 15 | 4 | 0.030514 | 35.00 |  |
| *CFAP44* | 14 | 3 | 0.030514 | 64.29 |  |
| *CUBN* | 28 | 4 | 0.030514 | 32.14 |  |
| *DDX60L* | 13 | 2 | 0.030514 | 30.77 |  |
| *DNAJC13* | 13 | 3 | 0.030514 | 23.08 |  |
| *FLT1* | 13 | 3 | 0.030514 | 23.08 | yes |
| *INSRR* | 10 | 3 | 0.030514 | -3.33 |  |
| *ITGA2* | 15 | 3 | 0.030514 | 40.00 |  |
| *KIAA0586* | 14 | 3 | 0.030514 | 42.86 |  |
| *KIAA2022* | 11 | 3 | 0.030514 | 36.36 |  |
| *LAMB4* | 11 | 3 | 0.030514 | 45.45 |  |
| *LIFR* | 11 | 3 | 0.030514 | 27.27 | yes |
| *MYH15* | 11 | 3 | 0.030514 | 27.27 |  |
| *NES* | 11 | 3 | 0.030514 | 36.36 |  |
| *NLRP11* | 12 | 3 | 0.030514 | 50.00 |  |
| *PCDHB4* | 10 | 3 | 0.030514 | 46.67 |  |
| *PCDHB5* | 11 | 3 | 0.030514 | 36.36 |  |
| *PPP2R2B* | 10 | 3 | 0.030514 | 16.67 |  |
| *SAMD9L* | 11 | 3 | 0.030514 | 45.45 |  |
| *SPEF2* | 12 | 4 | 0.030514 | 41.67 |  |
| *TBX18* | 13 | 3 | 0.030514 | 38.46 |  |
| *TEX14* | 11 | 3 | 0.030514 | 36.36 |  |
| *TOP2B* | 10 | 4 | 0.030514 | 30.00 |  |
| *ZNF148* | 10 | 3 | 0.030514 | 50.00 |  |
| *ZNF850* | 13 | 3 | 0.030514 | 76.92 |  |
| *CDH18* | 14 | 8 | 0.033984 | 50.00 | yes |
| *CNTNAP2* | 13 | 9 | 0.033984 | 46.15 |  |
| *FRY* | 20 | 10 | 0.033984 | 20.00 |  |
| *MYO18B* | 20 | 10 | 0.033984 | 45.00 |  |
| *TENM1* | 23 | 12 | 0.033984 | 26.45 |  |
| *WDFY4* | 35 | 8 | 0.033984 | 40.00 |  |
| *APC* | 41 | 15 | 0.040388 | 56.75 | yes (TSG) |
| *BFSP1* | 9 | 0 | 0.001273 | 66.67 |  |
| *SIK2* | 9 | 0 | 0.001273 | 44.44 |  |
| *USP26* | 9 | 0 | 0.001273 | 44.44 |  |
| *ANO2* | 9 | 1 | 0.001273 | 55.56 |  |
| *NUP107* | 9 | 1 | 0.001273 | -55.56 |  |
| *AFTPH* | 9 | 0 | 0.004004 | 55.56 |  |
| *ARHGAP5* | 9 | 0 | 0.004004 | 33.33 |  |
| *CHRM2* | 9 | 0 | 0.004004 | 22.22 |  |
| *PROX2* | 9 | 0 | 0.004004 | 33.33 |  |
| *TPTE2* | 9 | 0 | 0.004004 | 66.67 |  |
| *CCNB3* | 9 | 1 | 0.004004 | 66.67 |  |
| *SEMA3D* | 9 | 1 | 0.004004 | 66.67 |  |
| *ZFP28* | 9 | 1 | 0.004004 | 55.56 |  |
| *ADGRG6* | 9 | 2 | 0.008534 | 44.44 |  |
| *BTBD7* | 9 | 2 | 0.008534 | 5.56 |  |
| *CNGB3* | 9 | 2 | 0.008534 | 16.67 |  |
| *KDM3A* | 9 | 2 | 0.008534 | 55.56 |  |
| *PPP1R12B* | 9 | 2 | 0.008534 | 66.67 |  |
| *RAG1* | 9 | 2 | 0.008534 | 33.33 | yes |
| *RGSL1* | 9 | 2 | 0.008534 | 55.56 |  |
| *USP7* | 9 | 2 | 0.008534 | -38.89 |  |
| *ZNF160* | 9 | 2 | 0.008534 | 33.33 |  |
| *ABCF1* | 9 | 0 | 0.010764 | 0.00 |  |
| *EBF2* | 9 | 0 | 0.010764 | 22.22 |  |
| *FYN* | 9 | 0 | 0.010764 | 33.33 | yes |
| *HTR3B* | 9 | 0 | 0.010764 | 44.44 |  |
| *NME8* | 9 | 0 | 0.010764 | 22.22 |  |
| *PPIP5K2* | 9 | 0 | 0.010764 | 66.67 |  |
| *RMDN2* | 9 | 0 | 0.010764 | 44.44 |  |
| *RPS6KA5* | 9 | 0 | 0.010764 | 55.56 |  |
| *ZNF112* | 9 | 0 | 0.010764 | 77.78 |  |
| *ANKRD30B* | 9 | 1 | 0.010764 | 66.67 |  |
| *COBLL1* | 9 | 1 | 0.010764 | 33.33 |  |
| *ECT2* | 9 | 1 | 0.010764 | 77.78 |  |
| *FANCD2* | 9 | 1 | 0.010764 | 33.33 | yes |
| *HDX* | 9 | 1 | 0.010764 | 55.56 |  |
| *PIK3CB* | 9 | 1 | 0.010764 | 55.56 | yes |
| *SMC5* | 9 | 1 | 0.010764 | 55.56 |  |
| *SPAG1* | 9 | 1 | 0.010764 | 22.22 |  |
| *TEK* | 9 | 1 | 0.010764 | 11.11 |  |
| *ZNF790* | 9 | 1 | 0.010764 | 55.56 |  |
| *DENND5B* | 9 | 3 | 0.010764 | 33.33 |  |
| *PDZD8* | 9 | 2 | 0.012318 | 55.56 |  |
| *FAM179B* | 9 | 3 | 0.012318 | 22.22 |  |
| *GFPT1* | 9 | 3 | 0.012318 | 33.33 |  |
| *KCNB2* | 9 | 3 | 0.012318 | 11.11 |  |
| *ZNF546* | 9 | 3 | 0.012318 | 66.67 |  |
| *CSPP1* | 9 | 4 | 0.012318 | 55.56 |  |
| *ENPEP* | 9 | 4 | 0.012318 | 44.44 |  |
| *SLITRK5* | 9 | 4 | 0.012318 | 33.33 |  |
| *ADAMTS6* | 9 | 2 | 0.022867 | 5.56 |  |
| *ANKRD18B* | 9 | 2 | 0.022867 | 66.67 |  |
| *CLIP1* | 9 | 2 | 0.022867 | -5.56 | yes |
| *DNAI1* | 9 | 2 | 0.022867 | 44.44 |  |
| *FBXO43* | 9 | 2 | 0.022867 | 44.44 |  |
| *KCNA10* | 9 | 2 | 0.022867 | -5.56 |  |
| *KIDINS220* | 9 | 2 | 0.022867 | 33.33 |  |
| *NCKAP1L* | 9 | 2 | 0.022867 | -27.78 |  |
| *SLFN12L* | 9 | 2 | 0.022867 | 33.33 |  |
| *TRDN* | 9 | 2 | 0.022867 | 55.56 |  |
| *PCDH12* | 9 | 3 | 0.022867 | 44.44 |  |
| *PIWIL3* | 9 | 3 | 0.022867 | 33.33 |  |
| *MAGED1* | 9 | 4 | 0.023663 | 22.22 |  |
| *PDE5A* | 9 | 4 | 0.023663 | 30.56 |  |
| *UACA* | 9 | 4 | 0.023663 | 55.56 |  |
| *GRK4* | 9 | 0 | 0.027864 | 55.56 |  |
| *MAGEE2* | 9 | 0 | 0.027864 | 33.33 |  |
| *MPP4* | 9 | 0 | 0.027864 | 33.33 |  |
| *SIGLEC11* | 9 | 0 | 0.027864 | 33.33 |  |
| *CCDC102B* | 9 | 1 | 0.027864 | 11.11 |  |
| *DPP8* | 9 | 1 | 0.027864 | 55.56 |  |
| *ENTPD3* | 9 | 1 | 0.027864 | 33.33 |  |
| *EPB41L3* | 9 | 1 | 0.027864 | 44.44 |  |
| *EXOC6* | 9 | 1 | 0.027864 | 77.78 |  |
| *FAM135A* | 9 | 1 | 0.027864 | 11.11 |  |
| *FER* | 9 | 1 | 0.027864 | 55.56 |  |
| *KAT7* | 9 | 1 | 0.027864 | 66.67 |  |
| *MCF2L2* | 9 | 1 | 0.027864 | 66.67 |  |
| *MOV10L1* | 9 | 1 | 0.027864 | 44.44 |  |
| *NFATC3* | 9 | 1 | 0.027864 | 55.56 |  |
| *OR4A15* | 9 | 1 | 0.027864 | 22.22 |  |
| *PRKCE* | 9 | 1 | 0.027864 | 11.11 |  |
| *RALBP1* | 9 | 1 | 0.027864 | 44.44 |  |
| *SNX29* | 9 | 1 | 0.027864 | 55.56 | yes |
| *TDRD9* | 9 | 1 | 0.027864 | 33.33 |  |
| *TTC21A* | 9 | 1 | 0.027864 | 33.33 |  |
| *ZNF286B* | 9 | 1 | 0.027864 | 66.67 |  |
| *ZNF571* | 9 | 1 | 0.027864 | 55.56 |  |
| *CLSPN* | 9 | 2 | 0.027864 | 22.22 |  |
| *NFAT5* | 9 | 4 | 0.029543 | 33.33 |  |
| *GRM1* | 9 | 5 | 0.029543 | 33.33 |  |
| *WDR78* | 9 | 5 | 0.029543 | 22.22 |  |
| *CDK5RAP2* | 9 | 1 | 0.030514 | 55.56 |  |
| *ADAMTS1* | 9 | 2 | 0.030514 | 66.67 |  |
| *OTUD4* | 9 | 2 | 0.030514 | -16.67 |  |
| *CAPN14* | 9 | 3 | 0.030514 | 22.22 |  |
| *EHHADH* | 9 | 3 | 0.030514 | 66.67 |  |
| *KIAA1324L* | 9 | 3 | 0.030514 | 22.22 |  |
| *PKD2L1* | 9 | 3 | 0.030514 | 22.22 |  |
| *PLCB2* | 9 | 3 | 0.030514 | 77.78 |  |
| *RALGAPA2* | 9 | 3 | 0.030514 | 44.44 |  |
| *SIM1* | 9 | 3 | 0.030514 | 11.11 |  |
| *SLC6A17* | 9 | 3 | 0.030514 | 33.33 |  |
| *ZNF804A* | 9 | 3 | 0.030514 | 55.56 |  |
| *ADAMTS2* | 9 | 4 | 0.030514 | 44.44 |  |
| *ATP8B1* | 9 | 4 | 0.030514 | 30.56 |  |
| *CDKL5* | 9 | 4 | 0.030514 | 55.56 |  |
| *KCNH5* | 9 | 5 | 0.030514 | -20.00 |  |
| *MROH8* | 8 | 0 | 0.001273 | 50.00 |  |
| *PAPOLB* | 8 | 0 | 0.001273 | 62.50 |  |
| *CEP89* | 8 | 1 | 0.001273 | 62.50 | yes |
| *C1orf87* | 8 | 0 | 0.004004 | 25.00 |  |
| *F13B* | 8 | 0 | 0.004004 | 50.00 |  |
| *GAD2* | 8 | 0 | 0.004004 | 37.50 |  |
| *SLC9A2* | 8 | 0 | 0.004004 | 37.50 |  |
| *ZNF563* | 8 | 0 | 0.004004 | 100.00 |  |
| *C1orf101* | 8 | 1 | 0.004004 | 37.50 |  |
| *FRS2* | 8 | 1 | 0.004004 | 62.50 |  |
| *GYS2* | 8 | 1 | 0.004004 | 50.00 |  |
| *INPP4B* | 8 | 1 | 0.004004 | 62.50 | yes |
| *ZNF518B* | 8 | 1 | 0.008534 | 37.50 |  |
| *C14orf37* | 8 | 2 | 0.008534 | 37.50 |  |
| *TTF1* | 8 | 2 | 0.008534 | 0.00 |  |
| *CABS1* | 8 | 0 | 0.010764 | 50.00 |  |
| *DNTT* | 8 | 0 | 0.010764 | 37.50 |  |
| *FOXJ3* | 8 | 0 | 0.010764 | 37.50 |  |
| *KCNK10* | 8 | 0 | 0.010764 | 12.50 |  |
| *RBL1* | 8 | 0 | 0.010764 | 25.00 |  |
| *SENP5* | 8 | 0 | 0.010764 | 25.00 |  |
| *TMPRSS11F* | 8 | 0 | 0.010764 | 25.00 |  |
| *USP25* | 8 | 0 | 0.010764 | 62.50 |  |
| *ZFP37* | 8 | 0 | 0.010764 | 50.00 |  |
| *ZNF880* | 8 | 0 | 0.010764 | 50.00 |  |
| *ZNF883* | 8 | 0 | 0.010764 | 62.50 |  |
| *ZSCAN12* | 8 | 0 | 0.010764 | 50.00 |  |
| *CDC25C* | 8 | 1 | 0.010764 | 37.50 |  |
| *EXOC4* | 8 | 1 | 0.010764 | 62.50 |  |
| *HS6ST2* | 8 | 1 | 0.010764 | 25.00 |  |
| *OR5D16* | 8 | 1 | 0.010764 | 62.50 |  |
| *PKD1L3* | 8 | 1 | 0.010764 | 12.50 |  |
| *POU3F4* | 8 | 1 | 0.010764 | 50.00 |  |
| *PPEF1* | 8 | 1 | 0.010764 | 62.50 |  |
| *PROS1* | 8 | 1 | 0.010764 | 62.50 |  |
| *PTPN14* | 8 | 1 | 0.010764 | -75.00 |  |
| *SERPINB10* | 8 | 1 | 0.010764 | 37.50 |  |
| *USP29* | 8 | 1 | 0.010764 | 75.00 |  |
| *WDR64* | 8 | 1 | 0.010764 | -62.50 |  |
| *ZFP2* | 8 | 1 | 0.010764 | 100.00 |  |
| *LRRCC1* | 8 | 2 | 0.010764 | 37.50 |  |
| *PPP2R3A* | 8 | 3 | 0.012318 | 50.00 |  |
| *SLITRK3* | 8 | 5 | 0.012318 | 37.50 |  |
| *BCHE* | 8 | 2 | 0.022867 | 12.50 |  |
| *CAMSAP2* | 8 | 2 | 0.022867 | 37.50 |  |
| *F13A1* | 8 | 2 | 0.022867 | 62.50 |  |
| *FRMD7* | 8 | 2 | 0.022867 | 25.00 |  |
| *GUCY2C* | 8 | 2 | 0.022867 | 50.00 |  |
| *HFM1* | 8 | 2 | 0.022867 | 37.50 |  |
| *IQGAP1* | 8 | 2 | 0.022867 | -37.50 |  |
| *ITGA4* | 8 | 2 | 0.022867 | 37.50 |  |
| *LRFN5* | 8 | 2 | 0.022867 | 25.00 |  |
| *MARCH10* | 8 | 2 | 0.022867 | 62.50 |  |
| *MSR1* | 8 | 2 | 0.022867 | 12.50 |  |
| *MYO1H* | 8 | 2 | 0.022867 | 25.00 |  |
| *NCKAP5* | 8 | 2 | 0.022867 | 37.50 |  |
| *SV2C* | 8 | 2 | 0.022867 | 50.00 |  |
| *SYNGAP1* | 8 | 2 | 0.022867 | 37.50 |  |
| *TTLL5* | 8 | 2 | 0.022867 | 50.00 |  |
| *ZFP69B* | 8 | 2 | 0.022867 | 62.50 |  |
| *ZNF749* | 8 | 2 | 0.022867 | 50.00 |  |
| *MACC1* | 8 | 3 | 0.022867 | 50.00 |  |
| *N4BP2L2* | 8 | 3 | 0.022867 | 87.50 |  |
| *OPHN1* | 8 | 3 | 0.022867 | 50.00 |  |
| *ATAD2* | 8 | 4 | 0.023663 | 37.50 |  |
| *COL5A3* | 8 | 4 | 0.023663 | 50.00 |  |
| *SATB1* | 8 | 4 | 0.023663 | 50.00 |  |
| *C7orf72* | 8 | 0 | 0.027864 | 25.00 |  |
| *C9orf131* | 8 | 0 | 0.027864 | 25.00 |  |
| *CCDC181* | 8 | 0 | 0.027864 | 62.50 |  |
| *CLEC4F* | 8 | 0 | 0.027864 | 25.00 |  |
| *KIF6* | 8 | 0 | 0.027864 | 25.00 |  |
| *MTMR10* | 8 | 0 | 0.027864 | 37.50 |  |
| *NDUFS1* | 8 | 0 | 0.027864 | 62.50 |  |
| *NEDD1* | 8 | 0 | 0.027864 | 12.50 |  |
| *PUM2* | 8 | 0 | 0.027864 | 25.00 |  |
| *RAB3GAP1* | 8 | 0 | 0.027864 | 50.00 |  |
| *RANBP6* | 8 | 0 | 0.027864 | 37.50 |  |
| *RFX8* | 8 | 0 | 0.027864 | 50.00 |  |
| *SEC14L2* | 8 | 0 | 0.027864 | 25.00 |  |
| *TBC1D8B* | 8 | 0 | 0.027864 | 75.00 |  |
| *THEGL* | 8 | 0 | 0.027864 | 37.50 |  |
| *WDHD1* | 8 | 0 | 0.027864 | 50.00 |  |
| *ZNF763* | 8 | 0 | 0.027864 | 50.00 |  |
| *ZNF823* | 8 | 0 | 0.027864 | 50.00 |  |
| *ACE2* | 8 | 1 | 0.027864 | 50.00 |  |
| *DDX59* | 8 | 1 | 0.027864 | 12.50 |  |
| *FAM214A* | 8 | 1 | 0.027864 | 100.00 |  |
| *FETUB* | 8 | 1 | 0.027864 | 12.50 |  |
| *FEZ1* | 8 | 1 | 0.027864 | 62.50 |  |
| *FSTL5* | 8 | 1 | 0.027864 | 50.00 |  |
| *IQUB* | 8 | 1 | 0.027864 | 75.00 |  |
| *ITIH1* | 8 | 1 | 0.027864 | 25.00 |  |
| *MMEL1* | 8 | 1 | 0.027864 | 25.00 |  |
| *MTM1* | 8 | 1 | 0.027864 | 75.00 |  |
| *MX2* | 8 | 1 | 0.027864 | 62.50 |  |
| *NDST3* | 8 | 1 | 0.027864 | 25.00 |  |
| *PPIG* | 8 | 1 | 0.027864 | 25.00 |  |
| *QARS* | 8 | 1 | 0.027864 | 12.50 |  |
| *RALGPS1* | 8 | 1 | 0.027864 | 37.50 |  |
| *STAG2* | 8 | 1 | 0.027864 | 25.00 | yes (TSG) |
| *THADA* | 8 | 1 | 0.027864 | 12.50 |  |
| *TRO* | 8 | 1 | 0.027864 | 50.00 |  |
| *UGT8* | 8 | 1 | 0.027864 | 62.50 |  |
| *XRN1* | 8 | 1 | 0.027864 | 37.50 |  |
| *ZNF454* | 8 | 1 | 0.027864 | 50.00 |  |
| *ZNF479* | 8 | 1 | 0.027864 | 75.00 |  |
| *ZNF812P* | 8 | 1 | 0.027864 | 50.00 | yes |
| *MECOM* | 8 | 5 | 0.029543 | 55.00 | yes |
| *ITGAE* | 8 | 6 | 0.029543 | 37.50 |  |
| *PIWIL1* | 8 | 2 | 0.030514 | 0.00 |  |
| *ATP10B* | 8 | 3 | 0.030514 | 4.17 |  |
| *BRINP3* | 8 | 3 | 0.030514 | 37.50 |  |
| *KBTBD3* | 8 | 3 | 0.030514 | 75.00 |  |
| *KIAA1549* | 8 | 3 | 0.030514 | 50.00 | yes |
| *NCAM2* | 8 | 3 | 0.030514 | 62.50 |  |
| *RFX6* | 8 | 3 | 0.030514 | 4.17 |  |
| *RICTOR* | 8 | 3 | 0.030514 | 37.50 | yes |
| *SCAF11* | 8 | 3 | 0.030514 | 25.00 |  |
| *SYNE3* | 8 | 3 | 0.030514 | -20.83 |  |
| *CYP2A13* | 8 | 4 | 0.030514 | 25.00 |  |
| *GRIA4* | 8 | 4 | 0.030514 | 25.00 |  |
| *RANBP2* | 8 | 4 | 0.030514 | 25.00 | yes |
| *RAPGEF6* | 8 | 4 | 0.030514 | 37.50 |  |
| *DNAAF1* | 7 | 0 | 0.004004 | 71.43 |  |
| *KLF8* | 7 | 0 | 0.004004 | 28.57 |  |
| *KRT16* | 7 | 0 | 0.004004 | 57.14 |  |
| *MOSPD2* | 7 | 0 | 0.004004 | 14.29 |  |
| *PSD2* | 7 | 0 | 0.004004 | 28.57 |  |
| *TLR10* | 7 | 0 | 0.004004 | 57.14 |  |
| *TMEM26* | 7 | 0 | 0.004004 | 85.71 |  |
| *VAMP7* | 7 | 0 | 0.004004 | 28.57 |  |
| *ZNF331* | 7 | 0 | 0.004004 | 71.43 | yes |
| *ZNF624* | 7 | 0 | 0.004004 | 28.57 |  |
| *CNDP1* | 7 | 1 | 0.004004 | 42.86 |  |
| *GEN1* | 7 | 1 | 0.004004 | 57.14 |  |
| *PSG1* | 7 | 1 | 0.004004 | 14.29 |  |
| *COL28A1* | 7 | 1 | 0.008534 | 28.57 |  |
| *EPSTI1* | 7 | 2 | 0.008534 | -57.14 |  |
| *BCCIP* | 7 | 0 | 0.010764 | 42.86 |  |
| *CDKAL1* | 7 | 0 | 0.010764 | 42.86 |  |
| *EFCAB3* | 7 | 0 | 0.010764 | 28.57 |  |
| *IPMK* | 7 | 0 | 0.010764 | 28.57 |  |
| *LRRD1* | 7 | 0 | 0.010764 | 57.14 |  |
| *NOC3L* | 7 | 0 | 0.010764 | 57.14 |  |
| *OR2B2* | 7 | 0 | 0.010764 | 42.86 |  |
| *OR4C16* | 7 | 0 | 0.010764 | 42.86 |  |
| *SAMD3* | 7 | 0 | 0.010764 | 57.14 |  |
| *SLC40A1* | 7 | 0 | 0.010764 | 28.57 |  |
| *SLFN12* | 7 | 0 | 0.010764 | 42.86 |  |
| *SPZ1* | 7 | 0 | 0.010764 | 42.86 |  |
| *SV2B* | 7 | 0 | 0.010764 | 14.29 |  |
| *TMF1* | 7 | 0 | 0.010764 | 57.14 |  |
| *TTC37* | 7 | 0 | 0.010764 | 28.57 |  |
| *WAC* | 7 | 0 | 0.010764 | 42.86 | yes |
| *XPO5* | 7 | 0 | 0.010764 | 14.29 |  |
| *ZEB1* | 7 | 0 | 0.010764 | 0.00 |  |
| *ZFP30* | 7 | 0 | 0.010764 | 57.14 |  |
| *ZNF354B* | 7 | 0 | 0.010764 | 71.43 |  |
| *ZNF534* | 7 | 0 | 0.010764 | 42.86 |  |
| *ZNF681* | 7 | 0 | 0.010764 | 71.43 |  |
| *ZW10* | 7 | 0 | 0.010764 | 14.29 |  |
| *ADAM21* | 7 | 1 | 0.010764 | 28.57 |  |
| *ANO3* | 7 | 1 | 0.010764 | 57.14 |  |
| *BRDT* | 7 | 1 | 0.010764 | 57.14 |  |
| *FHAD1* | 7 | 1 | 0.010764 | 14.29 |  |
| *N4BP1* | 7 | 1 | 0.010764 | 42.86 |  |
| *NEK1* | 7 | 1 | 0.010764 | 28.57 |  |
| *PFKM* | 7 | 1 | 0.010764 | 28.57 |  |
| *PHEX* | 7 | 1 | 0.010764 | 42.86 |  |
| *MATN2* | 7 | 2 | 0.010764 | 85.71 |  |
| *MTTP* | 7 | 3 | 0.012318 | 42.86 |  |
| *UHRF1BP1L* | 7 | 3 | 0.012318 | 42.86 |  |
| *NRIP1* | 7 | 1 | 0.022867 | 28.57 |  |
| *ZNF569* | 7 | 1 | 0.022867 | 57.14 |  |
| *ARHGAP15* | 7 | 2 | 0.022867 | 42.86 |  |
| *C12orf56* | 7 | 2 | 0.022867 | -21.43 |  |
| *FASTKD3* | 7 | 2 | 0.022867 | 71.43 |  |
| *GNPTAB* | 7 | 2 | 0.022867 | 28.57 |  |
| *KIAA0753* | 7 | 2 | 0.022867 | -7.14 |  |
| *KIF5A* | 7 | 2 | 0.022867 | 28.57 |  |
| *SLC25A12* | 7 | 2 | 0.022867 | 57.14 |  |
| *TBC1D31* | 7 | 2 | 0.022867 | 28.57 |  |
| *XPR1* | 7 | 2 | 0.022867 | 28.57 |  |
| *ZNF605* | 7 | 2 | 0.022867 | 28.57 |  |
| *CHSY3* | 7 | 4 | 0.023663 | 42.86 |  |
| *PDE8B* | 7 | 4 | 0.023663 | 57.14 |  |
| *BEND2* | 7 | 0 | 0.027864 | 14.29 |  |
| *C1orf141* | 7 | 0 | 0.027864 | 28.57 |  |
| *CFAP26* | 7 | 0 | 0.027864 | 42.86 | yes |
| *IFI44* | 7 | 0 | 0.027864 | 57.14 |  |
| *KCNN2* | 7 | 0 | 0.027864 | 14.29 |  |
| *OPN3* | 7 | 0 | 0.027864 | 57.14 |  |
| *OR6C1* | 7 | 0 | 0.027864 | 42.86 |  |
| *PRDM4* | 7 | 0 | 0.027864 | 14.29 |  |
| *SLC2A12* | 7 | 0 | 0.027864 | 57.14 |  |
| *THOC1* | 7 | 0 | 0.027864 | 0.00 |  |
| *TRIM22* | 7 | 0 | 0.027864 | 57.14 |  |
| *TSHR* | 7 | 0 | 0.027864 | 14.29 | yes (Oncogene) |
| *VASH2* | 7 | 0 | 0.027864 | 14.29 |  |
| *ZFP90* | 7 | 0 | 0.027864 | 42.86 |  |
| *ZNF329* | 7 | 0 | 0.027864 | 71.43 |  |
| *ANKRD6* | 7 | 1 | 0.027864 | 57.14 |  |
| *CEP135* | 7 | 1 | 0.027864 | 57.14 |  |
| *CYBB* | 7 | 1 | 0.027864 | 42.86 |  |
| *CYLC1* | 7 | 1 | 0.027864 | 42.86 |  |
| *DDX20* | 7 | 1 | 0.027864 | 57.14 |  |
| *DZIP3* | 7 | 1 | 0.027864 | 57.14 |  |
| *FAP* | 7 | 1 | 0.027864 | -71.43 | yes |
| *GABRR1* | 7 | 1 | 0.027864 | 42.86 |  |
| *GLRA3* | 7 | 1 | 0.027864 | 28.57 |  |
| *GPR65* | 7 | 1 | 0.027864 | 28.57 |  |
| *GTF2A1L* | 7 | 1 | 0.027864 | 42.86 | yes |
| *HSF2* | 7 | 1 | 0.027864 | 42.86 |  |
| *HSPH1* | 7 | 1 | 0.027864 | 42.86 |  |
| *KCNA4* | 7 | 1 | 0.027864 | 28.57 |  |
| *KRT2* | 7 | 1 | 0.027864 | 14.29 |  |
| *MED1* | 7 | 1 | 0.027864 | 42.86 |  |
| *MLH3* | 7 | 1 | 0.027864 | 14.29 |  |
| *MPDZ* | 7 | 1 | 0.027864 | 14.29 |  |
| *RASSF2* | 7 | 1 | 0.027864 | 57.14 |  |
| *RASSF9* | 7 | 1 | 0.027864 | 14.29 |  |
| *RDX* | 7 | 1 | 0.027864 | 71.43 |  |
| *ZNF175* | 7 | 1 | 0.027864 | 71.43 |  |
| *ZNF727* | 7 | 1 | 0.027864 | 42.86 |  |
| *ZNF770* | 7 | 2 | 0.027864 | 14.29 |  |
| *CYP17A1* | 7 | 4 | 0.027864 | 42.86 | yes |
| *ZNF142* | 7 | 7 | 0.029543 | 28.57 |  |
| *NPR2* | 7 | 2 | 0.030514 | 0.00 |  |
| *STARD13* | 7 | 2 | 0.030514 | 28.57 |  |
| *CCDC136* | 7 | 3 | 0.030514 | 28.57 |  |
| *DPYD* | 7 | 3 | 0.030514 | -4.76 | yes |
| *GABRG1* | 7 | 3 | 0.030514 | 71.43 |  |
| *GON4L* | 7 | 3 | 0.030514 | 71.43 |  |
| *GPC5* | 7 | 3 | 0.030514 | 42.86 |  |
| *NELL2* | 7 | 3 | 0.030514 | 28.57 |  |
| *OR2F2* | 7 | 3 | 0.030514 | 23.81 |  |
| *SLC24A1* | 7 | 3 | 0.030514 | 42.86 |  |
| *SPAG17* | 7 | 3 | 0.030514 | 42.86 |  |
| *VRK1* | 7 | 3 | 0.030514 | -4.76 |  |
| *CASP12* | 6 | 0 | 0.004004 | 66.67 |  |
| *IMMP2L* | 6 | 0 | 0.004004 | 50.00 |  |
| *JRKL* | 6 | 0 | 0.004004 | 33.33 |  |
| *KPNA7* | 6 | 0 | 0.004004 | 66.67 |  |
| *MYF6* | 6 | 0 | 0.004004 | 33.33 |  |
| *SGCZ* | 6 | 0 | 0.004004 | 50.00 |  |
| *UGT2B4* | 6 | 0 | 0.004004 | 33.33 |  |
| *ZNF717* | 6 | 0 | 0.004004 | 50.00 |  |
| *AGGF1* | 6 | 0 | 0.010764 | 50.00 |  |
| *AGTR2* | 6 | 0 | 0.010764 | 50.00 |  |
| *ASB15* | 6 | 0 | 0.010764 | 83.33 |  |
| *CCDC15* | 6 | 0 | 0.010764 | 66.67 |  |
| *CCR4* | 6 | 0 | 0.010764 | 50.00 |  |
| *COPB1* | 6 | 0 | 0.010764 | 50.00 |  |
| *DAB2* | 6 | 0 | 0.010764 | 33.33 |  |
| *DNAJC27* | 6 | 0 | 0.010764 | 50.00 |  |
| *FAM217A* | 6 | 0 | 0.010764 | 0.00 |  |
| *FOXRED1* | 6 | 0 | 0.010764 | 33.33 |  |
| *GABRA2* | 6 | 0 | 0.010764 | 16.67 |  |
| *HKR1* | 6 | 0 | 0.010764 | 16.67 |  |
| *MPP5* | 6 | 0 | 0.010764 | 66.67 |  |
| *MYB* | 6 | 0 | 0.010764 | 33.33 | yes |
| *NDC80* | 6 | 0 | 0.010764 | 66.67 |  |
| *OLFML1* | 6 | 0 | 0.010764 | 83.33 |  |
| *OR10A5* | 6 | 0 | 0.010764 | 33.33 |  |
| *OR4F6* | 6 | 0 | 0.010764 | 33.33 |  |
| *OR4M2* | 6 | 0 | 0.010764 | 50.00 |  |
| *OR5K1* | 6 | 0 | 0.010764 | 33.33 |  |
| *OR8G5* | 6 | 0 | 0.010764 | 16.67 |  |
| *PHACTR3* | 6 | 0 | 0.010764 | 33.33 |  |
| *PLCD4* | 6 | 0 | 0.010764 | 33.33 |  |
| *SELP* | 6 | 0 | 0.010764 | 66.67 | yes |
| *SIRT5* | 6 | 0 | 0.010764 | 83.33 |  |
| *TIGD4* | 6 | 0 | 0.010764 | 66.67 |  |
| *TPH1* | 6 | 0 | 0.010764 | 50.00 |  |
| *ZIM3* | 6 | 0 | 0.010764 | 50.00 |  |
| *ZNF280A* | 6 | 0 | 0.010764 | 33.33 |  |
| *ZNF699* | 6 | 0 | 0.010764 | 83.33 |  |
| *ZNF713* | 6 | 0 | 0.010764 | 66.67 |  |
| *ALB* | 6 | 1 | 0.010764 | -50.00 |  |
| *FRMPD2* | 6 | 1 | 0.010764 | 66.67 |  |
| *LMBRD1* | 6 | 1 | 0.010764 | 16.67 |  |
| *NR5A2* | 6 | 1 | 0.010764 | 33.33 |  |
| *OR6C6* | 6 | 1 | 0.010764 | 50.00 |  |
| *PDP1* | 6 | 1 | 0.010764 | 33.33 |  |
| *SEMG2* | 6 | 1 | 0.010764 | 33.33 |  |
| *STEAP2* | 6 | 1 | 0.010764 | 66.67 |  |
| *STK3* | 6 | 1 | 0.010764 | 66.67 |  |
| *CROT* | 6 | 2 | 0.022867 | 16.67 |  |
| *ETAA1* | 6 | 2 | 0.022867 | 100.00 |  |
| *ITGA5* | 6 | 2 | 0.022867 | 33.33 |  |
| *LUZP4* | 6 | 2 | 0.022867 | 33.33 |  |
| *NEMF* | 6 | 2 | 0.022867 | 33.33 |  |
| *PDHA2* | 6 | 2 | 0.022867 | 33.33 |  |
| *PTPRR* | 6 | 2 | 0.022867 | 16.67 |  |
| *UGT1A7* | 6 | 2 | 0.022867 | 0.00 |  |
| *ZNF85* | 6 | 2 | 0.022867 | 50.00 |  |
| *RBMXL1* | 6 | 3 | 0.022867 | 50.00 |  |
| *ABCG5* | 6 | 0 | 0.027864 | 33.33 |  |
| *ACTC1* | 6 | 0 | 0.027864 | 0.00 |  |
| *ASB4* | 6 | 0 | 0.027864 | 16.67 |  |
| *BMP5* | 6 | 0 | 0.027864 | 66.67 |  |
| *C6orf10* | 6 | 0 | 0.027864 | 50.00 |  |
| *CDADC1* | 6 | 0 | 0.027864 | 66.67 |  |
| *CHN1* | 6 | 0 | 0.027864 | 33.33 | yes |
| *CLEC3A* | 6 | 0 | 0.027864 | 16.67 |  |
| *CLINT1* | 6 | 0 | 0.027864 | 50.00 |  |
| *CMAS* | 6 | 0 | 0.027864 | 50.00 |  |
| *CSDE1* | 6 | 0 | 0.027864 | 16.67 |  |
| *DDO* | 6 | 0 | 0.027864 | 33.33 |  |
| *ELL3* | 6 | 0 | 0.027864 | 16.67 |  |
| *FSIP1* | 6 | 0 | 0.027864 | 50.00 |  |
| *GANC* | 6 | 0 | 0.027864 | 50.00 |  |
| *GRK7* | 6 | 0 | 0.027864 | 16.67 |  |
| *HNRNPAB* | 6 | 0 | 0.027864 | 33.33 |  |
| *HTR1F* | 6 | 0 | 0.027864 | 50.00 |  |
| *JADE3* | 6 | 0 | 0.027864 | 33.33 |  |
| *MYBL1* | 6 | 0 | 0.027864 | 50.00 |  |
| *NCBP1* | 6 | 0 | 0.027864 | 0.00 |  |
| *NGLY1* | 6 | 0 | 0.027864 | 50.00 |  |
| *NOX4* | 6 | 0 | 0.027864 | 83.33 |  |
| *OR8J1* | 6 | 0 | 0.027864 | 33.33 |  |
| *P4HA2* | 6 | 0 | 0.027864 | 33.33 |  |
| *PRAMEF12* | 6 | 0 | 0.027864 | 33.33 |  |
| *SCFD2* | 6 | 0 | 0.027864 | 50.00 |  |
| *STK24* | 6 | 0 | 0.027864 | 33.33 |  |
| *SULT1E1* | 6 | 0 | 0.027864 | 16.67 |  |
| *TDRKH* | 6 | 0 | 0.027864 | 0.00 |  |
| *TMEM144* | 6 | 0 | 0.027864 | 16.67 |  |
| *TMEM232* | 6 | 0 | 0.027864 | 33.33 |  |
| *TRIP4* | 6 | 0 | 0.027864 | 16.67 |  |
| *TTBK2* | 6 | 0 | 0.027864 | 50.00 |  |
| *YES1* | 6 | 0 | 0.027864 | 50.00 | yes |
| *ZDHHC13* | 6 | 0 | 0.027864 | 50.00 |  |
| *ZNF189* | 6 | 0 | 0.027864 | 33.33 |  |
| *ZNF195* | 6 | 0 | 0.027864 | 50.00 |  |
| *ZNF354C* | 6 | 0 | 0.027864 | 33.33 |  |
| *ZNF391* | 6 | 0 | 0.027864 | 83.33 |  |
| *ZNF433* | 6 | 0 | 0.027864 | 50.00 |  |
| *ZNF555* | 6 | 0 | 0.027864 | 33.33 |  |
| *ZNF662* | 6 | 0 | 0.027864 | 66.67 |  |
| *ZNF878* | 6 | 0 | 0.027864 | 83.33 |  |
| *ADGRG7* | 6 | 1 | 0.027864 | 33.33 |  |
| *GPRASP2* | 6 | 1 | 0.027864 | 50.00 | yes |
| *BBS12* | 6 | 1 | 0.027864 | 33.33 |  |
| *C16orf59* | 6 | 1 | 0.027864 | 66.67 |  |
| *CCDC110* | 6 | 1 | 0.027864 | -50.00 |  |
| *CCDC175* | 6 | 1 | 0.027864 | 66.67 |  |
| *CUL2* | 6 | 1 | 0.027864 | 50.00 |  |
| *DRD3* | 6 | 1 | 0.027864 | 50.00 |  |
| *FAM13B* | 6 | 1 | 0.027864 | 33.33 |  |
| *FEZF1* | 6 | 1 | 0.027864 | -100.00 |  |
| *HBS1L* | 6 | 1 | 0.027864 | 16.67 |  |
| *HIPK2* | 6 | 1 | 0.027864 | 33.33 |  |
| *HNRNPU* | 6 | 1 | 0.027864 | 16.67 |  |
| *HPS5* | 6 | 1 | 0.027864 | 33.33 |  |
| *ITGAM* | 6 | 1 | 0.027864 | 66.67 |  |
| *KIZ* | 6 | 1 | 0.027864 | 66.67 |  |
| *LARP7* | 6 | 1 | 0.027864 | 83.33 |  |
| *LRIT3* | 6 | 1 | 0.027864 | 66.67 |  |
| *LRRC36* | 6 | 1 | 0.027864 | 33.33 |  |
| *MAP3K2* | 6 | 1 | 0.027864 | 66.67 |  |
| *MMRN2* | 6 | 1 | 0.027864 | 50.00 |  |
| *MRE11* | 6 | 1 | 0.027864 | 83.33 | yes |
| *NKAPL* | 6 | 1 | 0.027864 | -83.33 |  |
| *NUP153* | 6 | 1 | 0.027864 | 50.00 |  |
| *OR4K5* | 6 | 1 | 0.027864 | 50.00 |  |
| *PARP9* | 6 | 1 | 0.027864 | 16.67 |  |
| *POGZ* | 6 | 1 | 0.027864 | 50.00 |  |
| *SLC25A14* | 6 | 1 | 0.027864 | 33.33 |  |
| *SLC6A1* | 6 | 1 | 0.027864 | 16.67 |  |
| *PPP4R3A* | 6 | 1 | 0.027864 | 83.33 | yes |
| *SPDL1* | 6 | 1 | 0.027864 | 33.33 |  |
| *TMC2* | 6 | 1 | 0.027864 | -66.67 |  |
| *TMEM237* | 6 | 1 | 0.027864 | 33.33 |  |
| *TSSK1B* | 6 | 1 | 0.027864 | 83.33 |  |
| *TXNDC16* | 6 | 1 | 0.027864 | 50.00 |  |
| *UGT1A9* | 6 | 1 | 0.027864 | 50.00 |  |
| *UGT2B11* | 6 | 1 | 0.027864 | 50.00 |  |
| *USP1* | 6 | 1 | 0.027864 | 33.33 |  |
| *VPS35* | 6 | 1 | 0.027864 | 50.00 |  |
| *ZNF222* | 6 | 1 | 0.027864 | 33.33 |  |
| *ZNF443* | 6 | 1 | 0.027864 | 83.33 |  |
| *ZNF485* | 6 | 1 | 0.027864 | 83.33 |  |
| *ZNF800* | 6 | 1 | 0.027864 | 66.67 |  |
| *ZNF81* | 6 | 1 | 0.027864 | 66.67 |  |
| *ZNF813* | 6 | 1 | 0.027864 | 33.33 |  |
| *BCO2* | 6 | 2 | 0.027864 | 33.33 |  |
| *CDC40* | 6 | 2 | 0.027864 | 0.00 |  |
| *COL25A1* | 6 | 2 | 0.027864 | 33.33 |  |
| *DDR2* | 6 | 2 | 0.027864 | 50.00 | yes |
| *PLAA* | 6 | 2 | 0.027864 | 33.33 |  |
| *AMOT* | 6 | 2 | 0.030514 | 66.67 |  |
| *CHD1* | 6 | 3 | 0.030514 | 0.00 |  |
| *OR2M2* | 6 | 3 | 0.030514 | 33.33 |  |
| *OSBPL3* | 6 | 3 | 0.030514 | 33.33 |  |
| *PLD5* | 6 | 3 | 0.030514 | 66.67 |  |
| *THBS2* | 6 | 3 | 0.030514 | 0.00 |  |
| *ZNF648* | 6 | 3 | 0.030514 | 66.67 |  |
| *GPR149* | 6 | 5 | 0.030514 | 16.67 |  |
| *ACSM2A* | 5 | 0 | 0.010764 | 40.00 |  |
| *APOBEC3F* | 5 | 0 | 0.010764 | 60.00 |  |
| *ARL1* | 5 | 0 | 0.010764 | 80.00 |  |
| *BHMT* | 5 | 0 | 0.010764 | 40.00 |  |
| *CD300A* | 5 | 0 | 0.010764 | 40.00 |  |
| *CLEC9A* | 5 | 0 | 0.010764 | 20.00 |  |
| *CTNNA1* | 5 | 0 | 0.010764 | 80.00 | yes |
| *FAM209B* | 5 | 0 | 0.010764 | 60.00 |  |
| *FRMD5* | 5 | 0 | 0.010764 | 0.00 |  |
| *GPAM* | 5 | 0 | 0.010764 | 40.00 |  |
| *GRB14* | 5 | 0 | 0.010764 | 60.00 |  |
| *HELB* | 5 | 0 | 0.010764 | 20.00 |  |
| *HSD17B4* | 5 | 0 | 0.010764 | 20.00 |  |
| *IL18RAP* | 5 | 0 | 0.010764 | 20.00 |  |
| *IRAK3* | 5 | 0 | 0.010764 | 60.00 |  |
| *MTFR1* | 5 | 0 | 0.010764 | 40.00 |  |
| *MTRR* | 5 | 0 | 0.010764 | 40.00 | yes |
| *NAT8* | 5 | 0 | 0.010764 | 60.00 |  |
| *ODC1* | 5 | 0 | 0.010764 | 20.00 |  |
| *OR2T6* | 5 | 0 | 0.010764 | 40.00 |  |
| *OR5B2* | 5 | 0 | 0.010764 | 20.00 |  |
| *POLI* | 5 | 0 | 0.010764 | 40.00 |  |
| *SHISA6* | 5 | 0 | 0.010764 | 40.00 |  |
| *SLC4A1AP* | 5 | 0 | 0.010764 | 20.00 |  |
| *SNX2* | 5 | 0 | 0.010764 | 60.00 |  |
| *SULT2A1* | 5 | 0 | 0.010764 | 0.00 |  |
| *SUSD4* | 5 | 0 | 0.010764 | 40.00 |  |
| *TEX36* | 5 | 0 | 0.010764 | 60.00 |  |
| *TIGD2* | 5 | 0 | 0.010764 | 20.00 |  |
| *TM9SF3* | 5 | 0 | 0.010764 | 0.00 |  |
| *TOM1L2* | 5 | 0 | 0.010764 | 60.00 |  |
| *TRAF5* | 5 | 0 | 0.010764 | 40.00 |  |
| *TRIM49B* | 5 | 0 | 0.010764 | 60.00 |  |
| *TRNT1* | 5 | 0 | 0.010764 | 60.00 |  |
| *USP16* | 5 | 0 | 0.010764 | 60.00 |  |
| *VN1R1* | 5 | 0 | 0.010764 | 20.00 |  |
| *ZNF385D* | 5 | 0 | 0.010764 | 20.00 |  |
| *ACTR3* | 5 | 0 | 0.027864 | 20.00 |  |
| *ADGRF1* | 5 | 0 | 0.027864 | 20.00 |  |
| *AOAH* | 5 | 0 | 0.027864 | 40.00 |  |
| *ATP6V0D2* | 5 | 0 | 0.027864 | 80.00 |  |
| *BANK1* | 5 | 0 | 0.027864 | 60.00 |  |
| *C12orf40* | 5 | 0 | 0.027864 | 80.00 |  |
| *C14orf39* | 5 | 0 | 0.027864 | 40.00 |  |
| *CACUL1* | 5 | 0 | 0.027864 | 60.00 |  |
| *CAPNS2* | 5 | 0 | 0.027864 | 40.00 |  |
| *CD4* | 5 | 0 | 0.027864 | 0.00 |  |
| *CELF1* | 5 | 0 | 0.027864 | 0.00 |  |
| *CHIT1* | 5 | 0 | 0.027864 | 0.00 |  |
| *CPXCR1* | 5 | 0 | 0.027864 | 40.00 |  |
| *CXorf57* | 5 | 0 | 0.027864 | 40.00 |  |
| *CYHR1* | 5 | 0 | 0.027864 | 0.00 |  |
| *CYP4F12* | 5 | 0 | 0.027864 | 20.00 |  |
| *EFHB* | 5 | 0 | 0.027864 | 80.00 |  |
| *EIF4G2* | 5 | 0 | 0.027864 | 20.00 |  |
| *FAM227B* | 5 | 0 | 0.027864 | 20.00 |  |
| *FRK* | 5 | 0 | 0.027864 | 40.00 |  |
| *GC* | 5 | 0 | 0.027864 | 0.00 |  |
| *GLMN* | 5 | 0 | 0.027864 | 0.00 |  |
| *HLA-DRA* | 5 | 0 | 0.027864 | 20.00 |  |
| *HOXD10* | 5 | 0 | 0.027864 | 40.00 |  |
| *HTR2A* | 5 | 0 | 0.027864 | 40.00 |  |
| *IL23R* | 5 | 0 | 0.027864 | 60.00 |  |
| *IL6ST* | 5 | 0 | 0.027864 | 40.00 | yes |
| *IL7R* | 5 | 0 | 0.027864 | 80.00 | yes |
| *LNPK* | 5 | 0 | 0.027864 | 80.00 | yes |
| *LRRN2* | 5 | 0 | 0.027864 | 40.00 |  |
| *MAGEA12* | 5 | 0 | 0.027864 | 0.00 |  |
| *METTL16* | 5 | 0 | 0.027864 | 60.00 |  |
| *METTL21C* | 5 | 0 | 0.027864 | 40.00 |  |
| *MFN1* | 5 | 0 | 0.027864 | 20.00 |  |
| *MIA2* | 5 | 0 | 0.027864 | 40.00 |  |
| *MIOS* | 5 | 0 | 0.027864 | 40.00 |  |
| *MLLT3* | 5 | 0 | 0.027864 | 60.00 | yes |
| *MSI2* | 5 | 0 | 0.027864 | 20.00 | yes |
| *NCOA7* | 5 | 0 | 0.027864 | 20.00 |  |
| *NEK2* | 5 | 0 | 0.027864 | 20.00 |  |
| *NIPAL2* | 5 | 0 | 0.027864 | 20.00 |  |
| *OR2M5* | 5 | 0 | 0.027864 | 60.00 |  |
| *OR3A1* | 5 | 0 | 0.027864 | 80.00 |  |
| *OR4E2* | 5 | 0 | 0.027864 | 100.00 |  |
| *OR8I2* | 5 | 0 | 0.027864 | 80.00 |  |
| *OR8K1* | 5 | 0 | 0.027864 | 40.00 |  |
| *PABPC4* | 5 | 0 | 0.027864 | 60.00 |  |
| *PARP11* | 5 | 0 | 0.027864 | 80.00 |  |
| *PARP8* | 5 | 0 | 0.027864 | 40.00 |  |
| *PIH1D2* | 5 | 0 | 0.027864 | 20.00 |  |
| *PLEK* | 5 | 0 | 0.027864 | 40.00 |  |
| *PM20D1* | 5 | 0 | 0.027864 | 20.00 |  |
| *PSRC1* | 5 | 0 | 0.027864 | 80.00 |  |
| *PTCHD1* | 5 | 0 | 0.027864 | 20.00 |  |
| *RBAK* | 5 | 0 | 0.027864 | 60.00 |  |
| *RGPD4* | 5 | 0 | 0.027864 | 80.00 |  |
| *SENP2* | 5 | 0 | 0.027864 | 40.00 |  |
| *SEPT10* | 5 | 0 | 0.027864 | 20.00 |  |
| *SHC4* | 5 | 0 | 0.027864 | 20.00 |  |
| *SKAP1* | 5 | 0 | 0.027864 | 0.00 |  |
| *SLC15A5* | 5 | 0 | 0.027864 | 20.00 |  |
| *SLC22A1* | 5 | 0 | 0.027864 | 0.00 |  |
| *SLC35G1* | 5 | 0 | 0.027864 | 40.00 |  |
| *SLCO6A1* | 5 | 0 | 0.027864 | 20.00 |  |
| *SMG9* | 5 | 0 | 0.027864 | 0.00 |  |
| *KMT5B* | 5 | 0 | 0.027864 | 60.00 | yes |
| *TAS2R39* | 5 | 0 | 0.027864 | 40.00 |  |
| *TEX33* | 5 | 0 | 0.027864 | 40.00 |  |
| *TIMELESS* | 5 | 0 | 0.027864 | 40.00 |  |
| *TM4SF20* | 5 | 0 | 0.027864 | 60.00 |  |
| *TMEM5* | 5 | 0 | 0.027864 | 40.00 |  |
| *TMEM57* | 5 | 0 | 0.027864 | 60.00 |  |
| *TTC23L* | 5 | 0 | 0.027864 | 60.00 |  |
| *TUBGCP5* | 5 | 0 | 0.027864 | 80.00 |  |
| *ZNF14* | 5 | 0 | 0.027864 | 100.00 |  |
| *ZNF165* | 5 | 0 | 0.027864 | 40.00 |  |
| *ZNF280B* | 5 | 0 | 0.027864 | 60.00 |  |
| *ZNF285* | 5 | 0 | 0.027864 | 20.00 |  |
| *ZNF431* | 5 | 0 | 0.027864 | 40.00 |  |
| *ZNF736* | 5 | 0 | 0.027864 | 60.00 |  |
| *ZNF93* | 5 | 0 | 0.027864 | 0.00 |  |
| *ABCD2* | 5 | 1 | 0.027864 | 20.00 |  |
| *APPL1* | 5 | 1 | 0.027864 | 20.00 |  |
| *C2orf78* | 5 | 1 | 0.027864 | 60.00 |  |
| *CLEC1B* | 5 | 1 | 0.027864 | 40.00 |  |
| *CLECL1* | 5 | 1 | 0.027864 | 80.00 |  |
| *DDHD2* | 5 | 1 | 0.027864 | -80.00 |  |
| *ERAP2* | 5 | 1 | 0.027864 | 40.00 |  |
| *ERICH6* | 5 | 1 | 0.027864 | 40.00 |  |
| *ERO1A* | 5 | 1 | 0.027864 | 80.00 | yes |
| *IFIT1B* | 5 | 1 | 0.027864 | 80.00 |  |
| *INTS7* | 5 | 1 | 0.027864 | 60.00 |  |
| *LARP4* | 5 | 1 | 0.027864 | 40.00 |  |
| *MAMDC2* | 5 | 1 | 0.027864 | 40.00 |  |
| *NPFFR2* | 5 | 1 | 0.027864 | 60.00 |  |
| *OR8K5* | 5 | 1 | 0.027864 | 60.00 |  |
| *PACRG* | 5 | 1 | 0.027864 | 80.00 |  |
| *PLS1* | 5 | 1 | 0.027864 | 40.00 |  |
| *PTGIS* | 5 | 1 | 0.027864 | 0.00 |  |
| *RCN1* | 5 | 1 | 0.027864 | 60.00 |  |
| *RHOBTB3* | 5 | 1 | 0.027864 | 40.00 |  |
| *SAMD8* | 5 | 1 | 0.027864 | 20.00 |  |
| *SYCP2L* | 5 | 1 | 0.027864 | 20.00 |  |
| *TBCCD1* | 5 | 1 | 0.027864 | 20.00 |  |
| *TMPO* | 5 | 1 | 0.027864 | 60.00 |  |
| *TRIM60* | 5 | 1 | 0.027864 | 20.00 |  |
| *TXNRD3* | 5 | 1 | 0.027864 | 40.00 |  |
| *USP53* | 5 | 1 | 0.027864 | 60.00 |  |
| *ZMYM6* | 5 | 1 | 0.027864 | -80.00 |  |
| *ACOT2* | 4 | 0 | 0.027864 | 25.00 |  |
| *ACTR2* | 4 | 0 | 0.027864 | 25.00 |  |
| *ALG10* | 4 | 0 | 0.027864 | 25.00 |  |
| *ALG8* | 4 | 0 | 0.027864 | 75.00 |  |
| *AMY2B* | 4 | 0 | 0.027864 | 50.00 |  |
| *ANKH* | 4 | 0 | 0.027864 | 25.00 |  |
| *ANXA11* | 4 | 0 | 0.027864 | 50.00 |  |
| *APLF* | 4 | 0 | 0.027864 | 25.00 |  |
| *ARMCX5* | 4 | 0 | 0.027864 | 25.00 |  |
| *ATG16L1* | 4 | 0 | 0.027864 | 50.00 |  |
| *C10orf120* | 4 | 0 | 0.027864 | 0.00 |  |
| *C1orf27* | 4 | 0 | 0.027864 | 25.00 |  |
| *C1R* | 4 | 0 | 0.027864 | 25.00 |  |
| *C1S* | 4 | 0 | 0.027864 | 25.00 |  |
| *C21orf91* | 4 | 0 | 0.027864 | 75.00 |  |
| *CAMK2D* | 4 | 0 | 0.027864 | 75.00 |  |
| *CC2D2B* | 4 | 0 | 0.027864 | 0.00 |  |
| *CCSER2* | 4 | 0 | 0.027864 | 75.00 |  |
| *CDC16* | 4 | 0 | 0.027864 | 25.00 |  |
| *CDKL4* | 4 | 0 | 0.027864 | 50.00 |  |
| *CELF2* | 4 | 0 | 0.027864 | 25.00 |  |
| *CER1* | 4 | 0 | 0.027864 | 75.00 |  |
| *CHORDC1* | 4 | 0 | 0.027864 | 50.00 |  |
| *CPSF3* | 4 | 0 | 0.027864 | 25.00 |  |
| *CTCF* | 4 | 0 | 0.027864 | 25.00 | yes |
| *CTNNAL1* | 4 | 0 | 0.027864 | 25.00 |  |
| *CTPS1* | 4 | 0 | 0.027864 | 50.00 |  |
| *CTSE* | 4 | 0 | 0.027864 | 100.00 |  |
| *CXorf23* | 4 | 0 | 0.027864 | 50.00 |  |
| *CYP1B1* | 4 | 0 | 0.027864 | 75.00 | yes |
| *CYP3A4* | 4 | 0 | 0.027864 | 25.00 | yes |
| *CYP3A43* | 4 | 0 | 0.027864 | 50.00 |  |
| *DSTN* | 4 | 0 | 0.027864 | 75.00 |  |
| *DTWD1* | 4 | 0 | 0.027864 | 25.00 |  |
| *DYX1C1* | 4 | 0 | 0.027864 | 50.00 |  |
| *EFCAB9* | 4 | 0 | 0.027864 | 50.00 |  |
| *ELSPBP1* | 4 | 0 | 0.027864 | 50.00 |  |
| *ENTPD7* | 4 | 0 | 0.027864 | 75.00 |  |
| *EPS15* | 4 | 0 | 0.027864 | 25.00 | yes |
| *EXOC5* | 4 | 0 | 0.027864 | 0.00 |  |
| *FAM8A1* | 4 | 0 | 0.027864 | 75.00 |  |
| *FBXO16* | 4 | 0 | 0.027864 | 100.00 |  |
| *FKBP14* | 4 | 0 | 0.027864 | 75.00 |  |
| *FSTL1* | 4 | 0 | 0.027864 | 75.00 |  |
| *GGCT* | 4 | 0 | 0.027864 | 75.00 |  |
| *GSTCD* | 4 | 0 | 0.027864 | 100.00 |  |
| *HNRNPA2B1* | 4 | 0 | 0.027864 | 25.00 | yes |
| *HSPA4L* | 4 | 0 | 0.027864 | 50.00 |  |
| *IBSP* | 4 | 0 | 0.027864 | 75.00 |  |
| *IFT88* | 4 | 0 | 0.027864 | 75.00 |  |
| *KNSTRN* | 4 | 0 | 0.027864 | 50.00 | yes |
| *KRTAP13-3* | 4 | 0 | 0.027864 | 50.00 |  |
| *LIPJ* | 4 | 0 | 0.027864 | 50.00 |  |
| *LRRC28* | 4 | 0 | 0.027864 | 0.00 |  |
| *LRTM2* | 4 | 0 | 0.027864 | 25.00 |  |
| *LSAMP* | 4 | 0 | 0.027864 | 0.00 |  |
| *MAP2K4* | 4 | 0 | 0.027864 | 25.00 | yes (TSG(del)) |
| *MAS1* | 4 | 0 | 0.027864 | 50.00 |  |
| *MBD4* | 4 | 0 | 0.027864 | 0.00 |  |
| *METTL17* | 4 | 0 | 0.027864 | 25.00 |  |
| *MPP6* | 4 | 0 | 0.027864 | 50.00 |  |
| *MYCT1* | 4 | 0 | 0.027864 | 50.00 |  |
| *NBN* | 4 | 0 | 0.027864 | 50.00 | yes |
| *NSMAF* | 4 | 0 | 0.027864 | 25.00 |  |
| *NUBP1* | 4 | 0 | 0.027864 | 0.00 |  |
| *NUDC* | 4 | 0 | 0.027864 | 25.00 |  |
| *OR10T2* | 4 | 0 | 0.027864 | 50.00 |  |
| *OR1B1* | 4 | 0 | 0.027864 | 25.00 |  |
| *OR1S2* | 4 | 0 | 0.027864 | 25.00 |  |
| *OR56B1* | 4 | 0 | 0.027864 | 50.00 |  |
| *OR6C4* | 4 | 0 | 0.027864 | 75.00 |  |
| *OR7E24* | 4 | 0 | 0.027864 | 25.00 |  |
| *OR8A1* | 4 | 0 | 0.027864 | 50.00 |  |
| *OR9A4* | 4 | 0 | 0.027864 | 50.00 |  |
| *OSBPL1A* | 4 | 0 | 0.027864 | 25.00 |  |
| *PDC* | 4 | 0 | 0.027864 | 50.00 |  |
| *PDCD10* | 4 | 0 | 0.027864 | 75.00 |  |
| *PIGB* | 4 | 0 | 0.027864 | 25.00 |  |
| *PPP3CB* | 4 | 0 | 0.027864 | 100.00 |  |
| *PTK2* | 4 | 0 | 0.027864 | 50.00 |  |
| *PTTG1* | 4 | 0 | 0.027864 | 25.00 |  |
| *RASGEF1A* | 4 | 0 | 0.027864 | 25.00 |  |
| *RBM43* | 4 | 0 | 0.027864 | 50.00 |  |
| *RNF139* | 4 | 0 | 0.027864 | 75.00 |  |
| *RNF180* | 4 | 0 | 0.027864 | 25.00 |  |
| *RTP4* | 4 | 0 | 0.027864 | 50.00 |  |
| *SAXO2* | 4 | 0 | 0.027864 | 100.00 |  |
| *SCYL2* | 4 | 0 | 0.027864 | 75.00 |  |
| *SDCCAG8* | 4 | 0 | 0.027864 | 75.00 |  |
| *SLC16A1* | 4 | 0 | 0.027864 | 25.00 |  |
| *SLC27A3* | 4 | 0 | 0.027864 | 100.00 |  |
| *SLC30A10* | 4 | 0 | 0.027864 | 25.00 |  |
| *SNAP47* | 4 | 0 | 0.027864 | 50.00 |  |
| *SPATA6* | 4 | 0 | 0.027864 | 50.00 |  |
| *SRPK1* | 4 | 0 | 0.027864 | 25.00 |  |
| *TBC1D22A* | 4 | 0 | 0.027864 | 75.00 |  |
| *TDRD3* | 4 | 0 | 0.027864 | 50.00 |  |
| *TGFB2* | 4 | 0 | 0.027864 | 0.00 |  |
| *TPRG1* | 4 | 0 | 0.027864 | 25.00 |  |
| *TRIM23* | 4 | 0 | 0.027864 | 25.00 |  |
| *TULP3* | 4 | 0 | 0.027864 | 50.00 |  |
| *VCPIP1* | 4 | 0 | 0.027864 | 50.00 |  |
| *WDR61* | 4 | 0 | 0.027864 | 75.00 |  |
| *ZCCHC4* | 4 | 0 | 0.027864 | 25.00 |  |
| *ZNF100* | 4 | 0 | 0.027864 | 75.00 |  |
| *ZNF227* | 4 | 0 | 0.027864 | 75.00 |  |
| *ZNF33A* | 4 | 0 | 0.027864 | 50.00 |  |
| *ZNF467* | 4 | 0 | 0.027864 | 25.00 |  |
| *ZNF630* | 4 | 0 | 0.027864 | 100.00 |  |
| *ZNF639* | 4 | 0 | 0.027864 | 75.00 |  |

SNV, single nucleotide variation (non-synonymous); PS, propensity score; DDR, DNA damage repair; BER, Base excision repair; MMR, mismatch repair; NER, nucleotide excision repair; NHEJ, non-homologous end joining; TLS, translesion synthesis; HR, homologous Recombination; FA, Fanconi anaemia; FPC, fork protection complex; n.d., not determined. The *q*-value of POLE-category specific genes was calculated using the Fisher's exact test and Benjamini-Hochberg procedure. Cancer-related genes were extracted and curated from previous reports^2, 3, 4, 5, 6^ and public databases (Baylor Genetics, http://bmgl.com/; MD Anderson cancer center, https://www.mdanderson.org/; Myriad Genetics, https://myriad.com/; Foundation medicine, https://www.foundationmedicine.com/; MyCancerGenome, https://www.mycancergenome.org/; Johns Hopkins, http://www.hopkinsmedicine.org/kimmel_cancer_center/; Memorial Sloan Kettering Cancer Center, https://www.mskcc.org/; Dana-Farber Cancer Institute, http://www.dana-farber.org/; Oncomine® Cancer Research Panel, http://www.lifelabdx.com/pharma.php).

**Supplementary Table S2. List of samples referred to in a public repository**

| Identifier | Tissue | Repository name |
| --- | --- | --- |
| 587376 | Large intestine | COSMIC |
| HCC2998-H | Large intestine | COSMIC |
| HCC2998-L | Large intestine | COSMIC |
| TCGA-CA-6718-01 | Large intestine | COSMIC |
| 587222 | Large intestine | COSMIC |
| TCGA-AX-A05Z-01 | Endometrium | COSMIC |
| TCGA-BS-A0UF-01 | Endometrium | COSMIC |
| TCGA-BS-A0UV-01 | Endometrium | COSMIC |
| TCGA-AX-A0J0-01 | Endometrium | COSMIC |
| TCGA-B5-A0JY-01 | Endometrium | COSMIC |
| TCGA-D1-A17Q-01 | Endometrium | COSMIC |
| TCGA-D1-A16X-01 | Endometrium | COSMIC |
| TCGA-B5-A11N-01 | Endometrium | COSMIC |
| T155 | Endometrium | COSMIC |
| TCGA-AN-A046-01 | Breast | COSMIC |
| TCGA-AZ-4315-01 | Large intestine | COSMIC |
| sysucc-311T | Large intestine | COSMIC |
| TCGA-EI-6917-01 | Large intestine | COSMIC |
| T2269 | Large intestine | COSMIC |
| TCGA-AP-A056-01 | Endometrium | COSMIC |
| TCGA-B5-A11E-01 | Endometrium | COSMIC |
| TCGA-AP-A0LM-01 | Endometrium | COSMIC |
| TCGA-A5-A0GP-01 | Endometrium | COSMIC |
| TCGA-D1-A16Y-01 | Endometrium | COSMIC |
| TCGA-BR-8680-01 | Stomach | COSMIC |
| COAD-US | Colorectal | ICGC |
| UCEC-US | Uterus | ICGC |
| TCGA-B5-A0JY-10A-01D-A10O-09 | Endometrial (POLE category) | ICGC |
| TCGA-BS-A0UV-10A-01D-A10B-09 | Endometrial (POLE category) | ICGC |
| TCGA-BS-A0UF-10A-01D-A10B-09 | Endometrial (POLE category) | ICGC |
| TCGA-AX-A0J0-10A-02X-A10H-09 | Endometrial (POLE category) | ICGC |
| TCGA-AX-A05Z-10A-01W-A027-09 | Endometrial (POLE category) | ICGC |
| TCGA-D1-A17Q-10A-01D-A12J-09 | Endometrial (POLE category) | ICGC |
| TCGA-CA-6718-10A-01D-1835-10 | Colorectal (POLE category) | ICGC |
| TCGA-D1-A16X-10A-01D-A12J-09 | Endometrial (POLE category) | ICGC |
| TCGA-B5-A11N-10A-01D-A122-09 | Endometrial (POLE category) | ICGC |
| TCGA-AX-A0J1-10A-01W-A062-09 | endometrial | ICGC |
| TCGA-AM-5820-10A-01D-1650-10 | colorectal | ICGC |
| TCGA-AM-5821-10A-01D-1650-10 | colorectal | ICGC |
| TCGA-AA-3663-11A-01D-1719-10 | colorectal | ICGC |
| TCGA-BS-A0UJ-10A-01D-A127-09 | endometrial | ICGC |
| TCGA-AZ-6598-11A-01D-1771-10 | colorectal | ICGC |
| TCGA-D1-A167-10A-01D-A12J-09 | endometrial | ICGC |
| TCGA-D1-A15X-10A-01D-A122-09 | endometrial | ICGC |
| TCGA-AA-3492-11A-01D-1408-10 | colorectal | ICGC |
| TCGA-AZ-6601-11A-01D-1771-10 | colorectal | ICGC |
| TCGA-G4-6588-10A-01D-1771-10 | colorectal | ICGC |
| TCGA-A5-A0VP-10A-01D-A10B-09 | endometrial | ICGC |
| TCGA-G4-6628-10A-01D-1835-10 | colorectal | ICGC |
| TCGA-D5-6928-10A-01D-1924-10 | colorectal | ICGC |
| TCGA-A6-6781-10A-01D-1924-10 | colorectal | ICGC |
| TCGA-AD-6889-10A-01D-1924-10 | colorectal | ICGC |
| TCGA-A6-5665-10A-01D-1650-10 | colorectal | ICGC |
| TCGA-AD-6895-10A-01D-1924-10 | colorectal | ICGC |
| TCGA-F4-6570-10A-01D-1771-10 | colorectal | ICGC |
| TCGA-AY-6197-10A-01D-1719-10 | colorectal | ICGC |
| TCGA-AD-6964-10A-01D-1924-10 | colorectal | ICGC |
| TCGA-CK-5916-10A-01D-1650-10 | colorectal | ICGC |
| TCGA-AZ-4615-10A-01D-1408-10 | colorectal | ICGC |
| TCGA-CM-5861-10A-01D-1650-10 | colorectal | ICGC |
| TCGA-AD-5900-10A-01D-1650-10 | colorectal | ICGC |
| TCGA-AA-3713-11A-01D-1719-10 | colorectal | ICGC |
| TCGA-B5-A11Y-10A-01D-A10M-09 | endometrial | ICGC |
| TCGA-AP-A054-10A-01W-A062-09 | endometrial | ICGC |
| TCGA-D5-6930-10A-01D-1924-10 | colorectal | ICGC |
| TCGA-AU-6004-10A-01D-1719-10 | colorectal | ICGC |
| TCGA-D5-6540-10A-01D-1719-10 | colorectal | ICGC |
| TCGA-CM-6162-10A-01D-1650-10 | colorectal | ICGC |
| TCGA-G4-6586-10A-01D-1771-10 | colorectal | ICGC |
| TCGA-AX-A063-10A-01W-A027-09 | endometrial | ICGC |
| TCGA-B5-A11H-10B-01D-A122-09 | endometrial | ICGC |
| TCGA-A6-5661-10A-01D-1650-10 | colorectal | ICGC |
| TCGA-G4-6302-10A-01D-1719-10 | colorectal | ICGC |
| TCGA-CM-6171-10A-01D-1650-10 | colorectal | ICGC |
| TCGA-A6-6780-10A-01D-1835-10 | colorectal | ICGC |
| TCGA-F4-6856-10A-01D-1924-10 | colorectal | ICGC |
| TCGA-A6-6653-10A-01D-1771-10 | colorectal | ICGC |
| TCGA-G4-6320-10A-01D-1720-10 | colorectal | ICGC |
| TCGA-CM-4743-10A-01D-1719-10 | colorectal | ICGC |
| TCGA-B5-A11R-10A-01D-A122-09 | endometrial | ICGC |
| TCGA-D1-A177-10A-01D-A12J-09 | endometrial | ICGC |
| TCGA-D1-A174-10A-01D-A12J-09 | endometrial | ICGC |
| TCGA-CM-6674-10A-01D-1835-10 | colorectal | ICGC |
| TCGA-B5-A0K9-10A-02D-A10B-09 | endometrial | ICGC |

**Supplementary References**

1. Rosenthal R, McGranahan N, Herrero J, Taylor BS, Swanton C. DeconstructSigs: delineating mutational processes in single tumors distinguishes DNA repair deficiencies and patterns of carcinoma evolution. *Genome Biol* **17**, 31 (2016).

2. Nagashima T*, et al.* Optimizing an ion semiconductor sequencing data analysis method to identify somatic mutations in the genomes of cancer cells in clinical tissue samples. *Biomed Res* **37**, 359-366 (2016).

3. Ohshima K*, et al.* Integrated analysis of gene expression and copy number identified potential cancer driver genes with amplification-dependent overexpression in 1,454 solid tumors. *Sci Rep* **7**, 641 (2017).

4. Forbes SA*, et al.* COSMIC: exploring the world's knowledge of somatic mutations in human cancer. *Nucleic Acids Res* **43**, D805-811 (2015).

5. Shimoda Y*, et al.* Integrated next-generation sequencing analysis of whole exome and 409 cancer-related genes. *Biomed Res* **37**, 367-379 (2016).

6. Vogelstein B, Papadopoulos N, Velculescu VE, Zhou S, Diaz LA, Jr., Kinzler KW. Cancer genome landscapes. *Science* **339**, 1546-1558 (2013).
